# Transcriptome analysis reveals the clinical significance of CXCL13 in Pan-Gyn tumors

**DOI:** 10.1007/s00432-024-05619-3

**Published:** 2024-03-09

**Authors:** Yue Ding, Quan Zhou, Bo Ding, Yang Zhang, Yang Shen

**Affiliations:** 1 Zhongda Hospital Southeast University, Nanjing, China; 2 Department of Obstetrics and Gynecology, First People’s Hospital of Lianyungang, No. 6 East Zhenhua Road, Haizhou Lianyungang, China

**Keywords:** CXCL13, Pan-Gyn, Immune infiltrate, Nomogram

## Abstract

**Background:**

Gynecologic and breast tumors (Pan-Gyn) exhibit similar characteristics, and the role of CXCL13 in anti-tumor immunity and it’s potential as a biomarker for immune checkpoint blockade (ICB) therapy have been gradually revealed. However, the precise role of CXCL13 in Pan-Gyn remains unclear, lacking a systematic analysis.

**Methods:**

We analyzed 2497 Pan-Gyn samples from the TCGA database, categorizing them into high and low CXCL13 expression groups. Validation was conducted using tumor expression datasets sourced from the GEO database. Correlation between CXCL13 and tumor immune microenvironment (TIME) was evaluated using multiple algorithms. Finally, we established nomograms for 3-year and 5-year mortality.

**Results:**

High expression of CXCL13 in Pan-Gyn correlates with a favorable clinical prognosis, increased immune cell infiltration, and reduced intra-tumor heterogeneity. Model was assessed using the C-index [BRCA: 0.763 (0.732–0.794), UCEC: 0.821 (0.793–0.849), CESC: 0.736 (0.684–0.788), and OV: 0.728 (0.707–0.749)], showing decent prediction of discrimination and calibration.

**Conclusion:**

Overall, this study provides comprehensive insights into the commonalities and differences of CXCL13 in Pan-Gyn, potentially opening new avenues for personalized treatment.

**Supplementary Information:**

The online version contains supplementary material available at 10.1007/s00432-024-05619-3.

## Introduction

Gynecological tumors are a primary contributors to cancer-related mortality among women worldwide (Zhu et al. 2018). Not coincidentally, breast cancer is the most common cancer and also a significant contributor to disease burden worldwide (Husby et al. 2018). Breast cancer was consistent with and similar to gynecological tumors (cervical cancer, ovarian cancer, and endometrial carcinoma) in multiple ways (Chung et al. 2020; Harris et al. 2020; Kalaitzopoulos et al. 2020; Xu et al. 2021). In this study, gynecologic tumors and breast cancer were defined as Pan-Gyn tumors.

Pan-Gyn tumors share lots of characteristics: they originate from the same embryonic sources in the Müllerian ducts, undergo development under the influence of female hormones, and share commonalities at the molecular level (Berger et al. 2018). For the majority of patients, first line of treatment consists of conventional methods such as surgery, chemotherapy, and radiotherapy (Cannistra and Pujade-Lauraine 2019; Trayes and Cokenakes 2021). More and more attention has been paid to the application of immune checkpoint inhibitors (ICIs) and PARP inhibitors (PARPi) in solid tumors now (Morad et al. 2021; Vanacker et al. 2021). DNA damage repair pathways have been focal points for therapy in most tumors (Lee et al. 2017). For example, PARPi has received approval as a standalone treatment for individuals with BRCA-mutated ovarian and breast cancer, which also includes TNBC (Verma et al. 2020). ICIs have established themselves as the therapy norm for many solid cancers recently because of the certain anti-tumor potential (Ritchie et al. 2018). However, a monotherapy approach will yield benefits for a small portion of patients. In addition, although PARPi is more widely used than ICIs in gynecological tumors, the current biomarkers still cannot accurately identify the applicable patients (Wagener-Ryczek et al. 2021). Moreover, a variety of challenges need to be overcome to appropriately select patients by valuable objective markers. Existing studies have confirmed the indispensable role of CXCL13 in ovarian cancer and can act as a biomarker (Yang et al. 2021), but it remains unclear in other gynecologic tumors and breast cancer.

CXCL13 is a cytokine secreted by stromal cells in the B-cell region of secondary lymphoid tissue (Cosgrove et al. 2020). It strongly attracts B lymphocytes and plays an important role in regulating the spatial migration of secondary lymphocytes (Hsieh et al. 2022a). Utilizing its receptor CXCR5, CXCL13 governs the movement of these cells into secondary lymphoid organs following the CXCL13 gradient and is marked by high expression on circulating follicular helper T cells, B lymphocytes, and skin-derived dendritic cells (Rubio et al. 2020). Our previous work confirms that CXCL13 is not only a biomarker that can be used for ICIs therapy but also a marker to evaluate the feasibility of immunotherapy for homologous recombination deficiency patients who respond poorly to PARPi. Up until now, investigations concerning CXCL13 within tumor contexts have been constrained to distinct types, like ovarian cancer. Therefore, it is imperative to analyze the target gene in Pan-Gyn, assessing its connection to fundamental molecular mechanisms, clinical phenotypic traits, and TIME. Thus far, PARPi and ICIs have revolutionized the treatment of tumors and have a wide range of application prospects in Pan-Gyn tumors (Pham et al. 2021). Therefore, it is necessary to elucidate the role of CXCL13 and commonalities in Pan-Gyn.

We try to thoroughly evaluate the expression of CXCL13 in Pan-Gyn based on the bioinformatics analysis to explore the relationship between expression levels and TIME. Hence, in the following study, our attention is directed toward the subsequent four TCGA cancer types: invasive breast carcinoma (BRCA), cervical squamous cell carcinoma and endocervical adenocarcinoma (CESC), uterine corpus endometrial carcinoma (UCEC), and ovarian cystadenocarcinoma (OV). Nomograms offer a more intuitive, elegant, and efficient means of presenting risk model outcomes, providing a convenient tool for predicting clinical results (Yin et al. 2021). So, the prognostic nomograms for Pan-Gyn patients were established based on CXCL13 gene data and clinical parameters identified using a Cox regression which was used to determine potentially important prognostic factors. Furthermore, we explored the potential mechanism of CXCL13 concerning the occurrence and development of Pan-Gyn. This study was pioneering in conducting a Pan-Gyn analysis based on CXCL13 expression and revealed that CXCL13 can serve as a prognostic factor, concurrently playing a pivotal role in TIME by influencing tumor-infiltrating immune cells and apoptotic pathways. In detail, we focused on elucidating different expressions, immune characteristics, and potential clinical application of CXCL13.

## Materials and methods

### Data collection and pre-processing

We obtained RNA sequencing data and correlated clinical follow-up information from the TCGA database, which encompasses 2497 samples. In addition, we obtained BRCA, CESC, and OV for validation sets from GEO database. RNA-seq data in FPKM format are converted to TPM format, then the value uses log_2_ transformation. Duplicate samples were removed while clinical information was retained. Somatic mutation data were acquired from the cBioPortal database (http://www.cbioportal.org/study/summary).

### Protein structures and interaction networks

The visualization of proteins plays a pivotal role in enhancing the presentation of results, promoting the generation of hypotheses, and facilitating logical reasoning regarding molecular structure (Martinez et al. 2020). Visualization of CXCL13 protein structures was performed by the UniProt (https://www.uniprot.org/) and Protein Data Bank (PDB) database (https://www.wwpdb.org/). In addition, STRING website (https://cn.string-db.org) serves as an interactive and user-friendly platform for constructing a protein–protein interaction (PPI) network, which is crucial for gaining improved insights into the functions and effectiveness of CXCL13. Hence, we used STRING database to generate PPI network of CXCL13 with minimum required interaction score 0.7 and the interaction predictions were mainly derived from experiments and databases.

### Correlation analysis between the expression levels of CXCL13 and survival outcomes

We obtained RNA-seq data for OV patients from the TCGA database, while normal samples were acquired from the GTEx project through UCSC Xena. Kaplan–Meier survival analysis was used to illustrate the relevancy between CXCL13 and patient prognosis. Overall survival (OS), disease-specific survival (DSS), and progression-free interval (PFI) are considered as the indicators of prognosis.

### Genomic alterations and methylation

Alteration of CXCL13 status was sourced from the cBioPortal database. Genomic alterations in CXCL13 included copy number amplification, missense mutation, and deep deletion.

### Tumor stemness

Tumor stemness scores were obtained from the web database of Sangerbox (http://vip.sangerbox.com/home.html). We intersected CXCL13 with stemness score (RNA expression-based, RNAss) to perform the Spearman correlation analysis. We also examined the overlap between CXCL13 and the stemness score (DNA methylation–based, DNAss) to conduct the Spearman correlation.

### Immuno-infiltration analysis

TIMER was used to analyze the relationship between immune cell infiltration and regulation of gene expression (Li et al. 2020). TIMER determined the proportional presence of six immune cell types for each sample. In addition, the relative infiltration ratios of eight immune cell types were obtained by the EPIC method (Racle et al. 2017). Furthermore, CIBERSORT (Newman et al. 2015) was used to calculate the relative scores, enabling the prediction of immunocyte phenotypes. Moreover, the correlations between CXCL13 and each immune cell infiltration level were also assessed using additional algorithms, including xCell, EPIC, QUANTISEQ, and MCPcounter. Correlational analyses were performed employing Spearman’s correlation. *p* < 0.05 and | *r* |> 0.3 were established as the cutoff criteria, and | *r* |> 0.6 indicate a strong correlation.

### Gene set enrichment analysis (GSEA)

GSEA was performed using R packages “cluster Profiler” in the high-expression and low-expression groups of CXCL13. The top five pathways were exhibited. The enrichment findings were presented using the net enrichment score (NES), FDR, and *p* value. Gene sets with |NES|> 1, adjust-*p* < 0.05, and FDR *q* < 0.25 met the criteria for significance.

### Immunohistochemical staining of CXCL13

The Human Protein Atlas project (https://www.proteinatlas.org/) is a commonly utilized proteome atlas database, which integrates data from various omics technologies to provide information on the protein distribution within tissues and cells. To compare differential expression of CXCL13, we downloaded immunohistochemical images of Pan-Gyn with corresponding normal tissues.

### Single-cell transcriptome analysis

Single-cell transcriptome analysis of pre-existing data was performed using the online public databases: Tumor Immune Single-cell Hub 2 (TISCH2, http://tisch.comp-genomics.org/home/). TISCH2 provides detailed cell-type annotation, facilitating the study of TIME across various cancer types (Sun et al. 2021). We calculated the gene–gene correlation between CXCL13 and some immune-related genes (PDCD1, CTLA4, CD8A, CD4, MS4A1), as well as the relationship with apoptosis. Apoptotic signaling pathway analyses were done by GSEA.

### Construction and verification of nomogram

Variables screened by univariate analysis with *p* values ≤ 0.1 were entered into the multivariate COX regression. If a significant *p* value (*p* < 0.05) was observed, independent prognostic factors were determined. Nomograms were constructed using significant clinical features from the final regression analysis. We evaluated the discriminative ability of the nomograms using the C-index. The more the C-index approached, the more accurate the predicted results of the nomogram. In addition, calibration of the nomograms was performed by visual calibration curves.

### Statistics

The Chi-square test was employed for comparing categorical variables. The correlation analysis was evaluated using Spearman’s correlation analysis. The R software (version 4.1) was utilized for all analyses. Statistical analyses were performed with the “rms” package. The R packages “ggplot2” and “ggpubr” were used to visualize results. FDR was used to correct for p values.

## Results

### The molecular structures and PPI networks of CXCL13

**Fig. 1:**
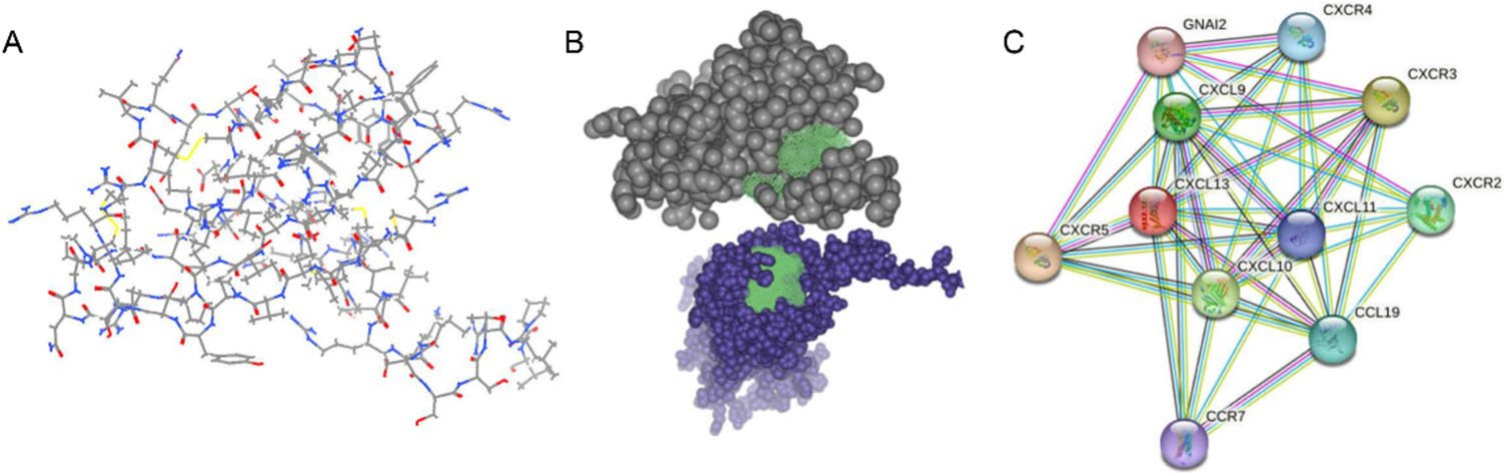
Protein structure and PPI networks of CXCL13.** A** The structure of CXCL13 protein; **B** The potential binding domain of CXCL13/CXCR5; **C** PPI network of CXCL13

The human CXCL13 protein contains 109 amino acids while C-X-C chemokine receptor type 5 (CXCR5) is composed of 372 amino acids. Protein structure was downloaded from the PDB website (Fig. 1A). The CXCL13 tertiary structure consists of an unstructured N-terminal ‘signaling domain’ succeeded by a ‘core domain’ and a C-terminal α-helix. In the crystal structure of human CXCL13, the initial methionine was observed to engage with the ‘core domain’ of CXCL13, potentially providing stability to the N-terminal structure (Pan et al. 2022). Actually, CXCL13 demonstrates considerable flexibility in its N-terminal region, while the core domain of the chemokine remains predominantly unaltered (Rosenberg et al. 2020). In addition, a remarkably extended and disordered C-terminal domain harboring a large degree of flexibility was also observed in the CXCL13 protein. CXCL13 exclusively binds CXCR5, which serves as a pivotal factor in the activation and mobilization of immune cells, and the modulation of the adaptive immune response (Hsieh et al. 2022b). Human CXCR5 is a seven-transmembrane G protein-coupled receptor, and it exists in two isoforms as a result of alternatively spliced transcript variants (Monneau et al. 2017). Differentiating from the predominant transcript variant 1, transcript variant 2 exhibits a variation at the 5’ end, which leads to translation initiation from a downstream in-frame AUG, consequently yielding an isoform with a shortened N-terminus (Pan et al. 2022). Yet, there is limited knowledge regarding the crystal structure of CXCR5. Similarly, there is a paucity of research on the structural foundation of CXCL13-CXCR5 interactions. We predicted a putative protein interaction site of CXCL13-CXCR5 by online tool (http://www.yinfotek.com/) (Fig. 1B). The PPI network of CXCL13 has been constructed by STRING, encompassing both confirmed and predicted protein–protein interactions, in both physical and functional aspects. The PPI networks show that CXCL13 mainly interacts with immune-related proteins and cell cycle-related proteins (Fig. 1C).

### Genetic alteration and methylation

**Fig. 2:**
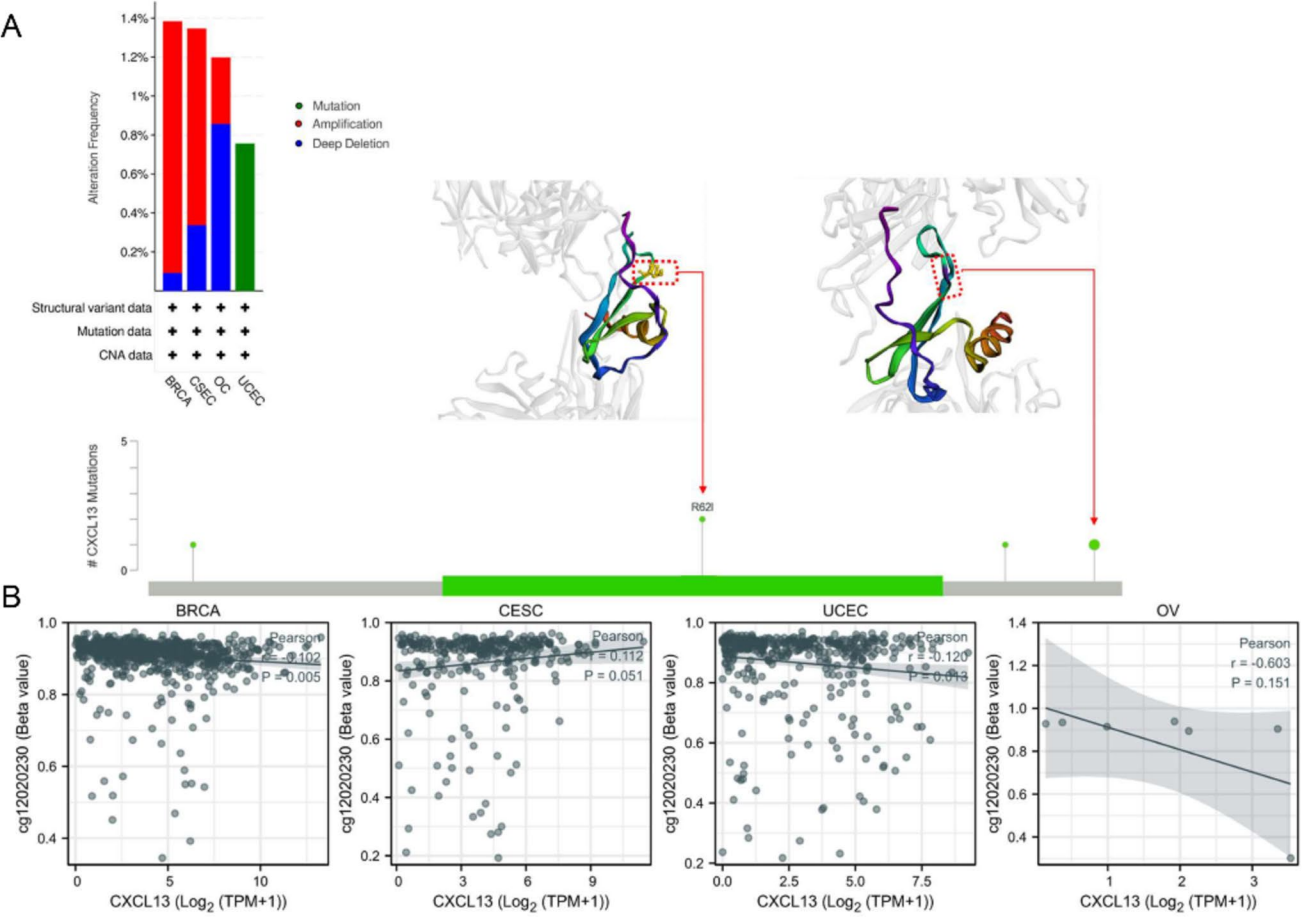
DNA methylation and mutation features of CXCL13 in Pan-Gyn.** A** Alteration frequency with mutation type and mutation site of CXCL13; **B** The correlation between CXCL13 expression and methylation level in Pan-Gyn (Pearson correlation)

Aberrant methylation and alteration in cancer cells mostly contribute to tumor progression and cancer cell plasticity (Ilango et al. 2020; Singh et al. 2022). Thus, we further explore the CXCL13 genetic alterations and epigenetics changes in Pan-Gyn. According to our analysis, the alteration frequency of CXCL13 is the highest in UCEC with “mutation” as the primary type (Fig. 2A). BRCA and CESC had the highest incidence of “amplification” while OC with “Deep Deletion”. We also acquired the mutation site visualized on the 3D structure of CXCL13 protein. We explored the relevancy of CXCL13 expression and methylation among all four tumors. Furthermore, we noticed that the expression of CXCL13 is positively correlated in CESC, whereas is negatively correlated with methylation in BRCA, UCEC, and OC (Fig. 2B). In addition to these, we also assess gene mutation between two groups (CXCL13-H/CXCL13-L). Top15 variants with high variation frequencies and significant differences between the two groups are shown in Figs. S1–S2.

### Differential expression and survival analysis of the CXCL13 in Pan-Gyn

**Fig. 3:**
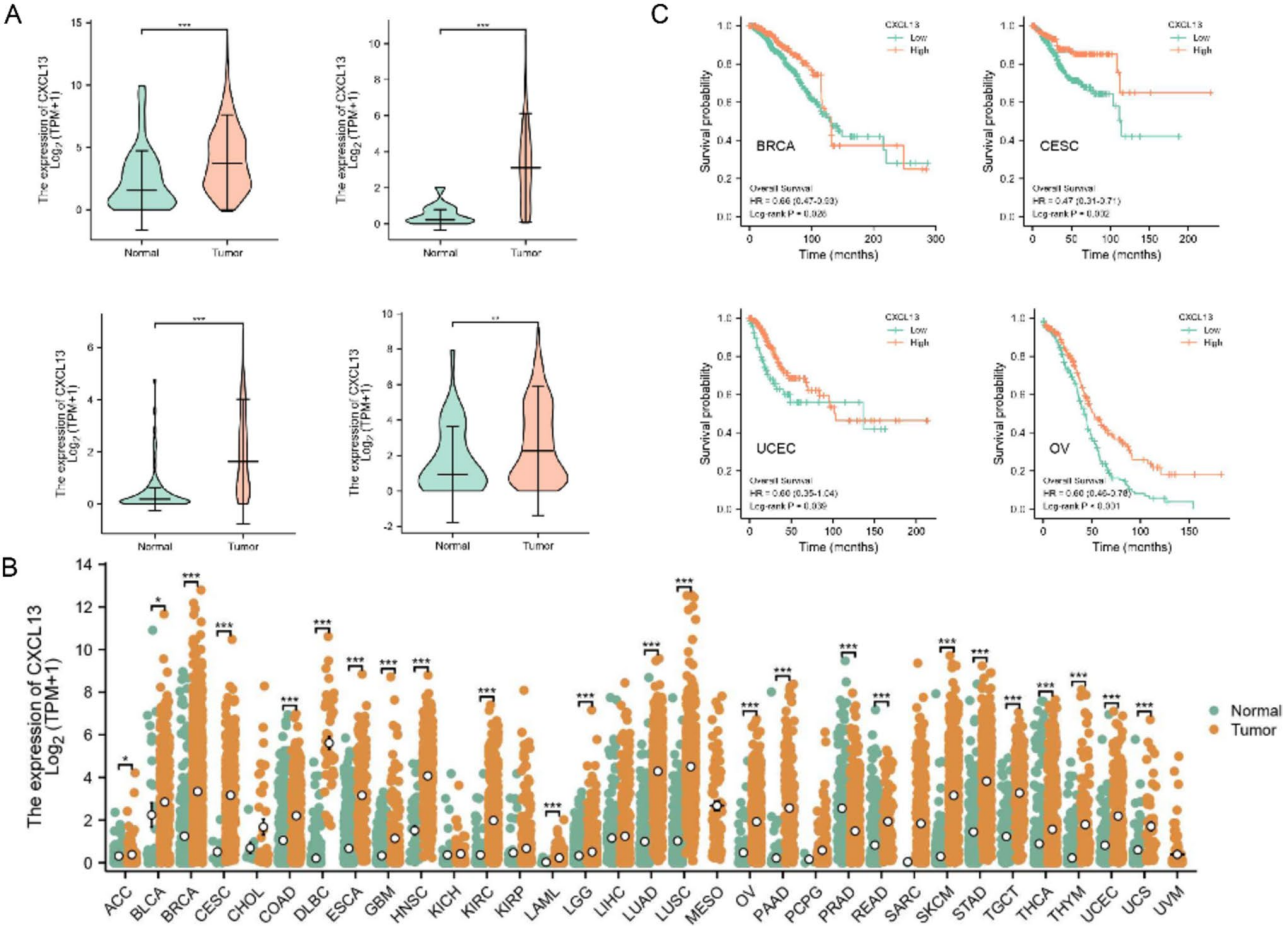
Differential expression and survival analysis of CXCL13 in Pan-Gyn.** A** CXCL13 expression in different cancers and paired normal tissue (clockwise direction: BRCA; CESC; OV; UCEC); **B** Expression of CXCL13 in pan-cancers using the TCGA database; **C** Kaplan–Meier curves showing the impact of CXCL13 expression on overall survival (clockwise direction: BRCA; CESC; OV; UCEC). *p < 0.05, **p < 0.01, ***p < 0.001

As normal controls were not available from TCGA, we integrated the GTEx database, which includes normal ovarian samples, to conduct the analysis of CXCL13’s differential expression. Violin plots show that expression of CXCL13 was significantly higher in tumors compared to the para-cancerous/normal tissues (Fig. 3A), and similar conclusions were obtained at the pan-cancer level (Fig. 3B). In addition, we examined CXCL13 protein levels using the immunohistochemistry results from HPA database and a similar conclusion was obtained (Fig. S3A). With the goal of exploring the connection between the expression level of CXCL13 and prognosis, we conducted survival analysis in Pan-Gyn, with a specific focus on OS, DSS, and PFI. For OS, the survival time was better for patients with high expression of CXCL13 (Fig. 3C log-rank *p* < 0.05) and we used GEO data for further validation (Fig. S7C). Kaplan–Meier survival analysis also demonstrated that among patients with OV, UCEC, and CESC, high CCL13 expression was associated with better DFS and PFI, while in patients with BRCA, those with high CXCL13 expression had no significant difference shown (Fig. S3B-C). These findings suggest that patients with high expression of CXCL13 had a satisfactory prognosis.

### Relationship between CXCL13 and tumor stemness

Tumor stemness, which denotes cancer cells’ stem-cell-like characteristics, is well established as a crucial factor in the initiation of tumorigenesis (Xiong et al. 2021). In general, elevated stemness score can reflect tumor progression. In Pan-Gyn, correlations between the levels of CXCL13 and RNAss are weakly negative in OV (Spearman *r* = − 0.102) and CESC (Spearman *r* = − 0.04) while weakly positive correlation in UCEC (Spearman *r* = 0.04) and BRCA (Spearman *r* = 0.094), as shown in Fig S3 D. Meanwhile, the expression level of CXCL13 was negatively correlated with DNAss in UCEC (Spearman *r* = − 0.21) and BRCA (Spearman *r* = − 0.02), while positively correlated in OV (Spearman *r* = 0.67). The result only shows a good agreement between CXCL13 and tumor stemness score in ovarian cancer. From the correlation, CXCL13 might not reflect the tumor stemness well in Pan-Gyn. Altogether, we need to further explore correlations between stemness score and CXCL13 (not as a single biomarker) to reveal the complexity and functionality.

### GSEA enrichment analysis in Pan-Gyn

**Fig. 4:**
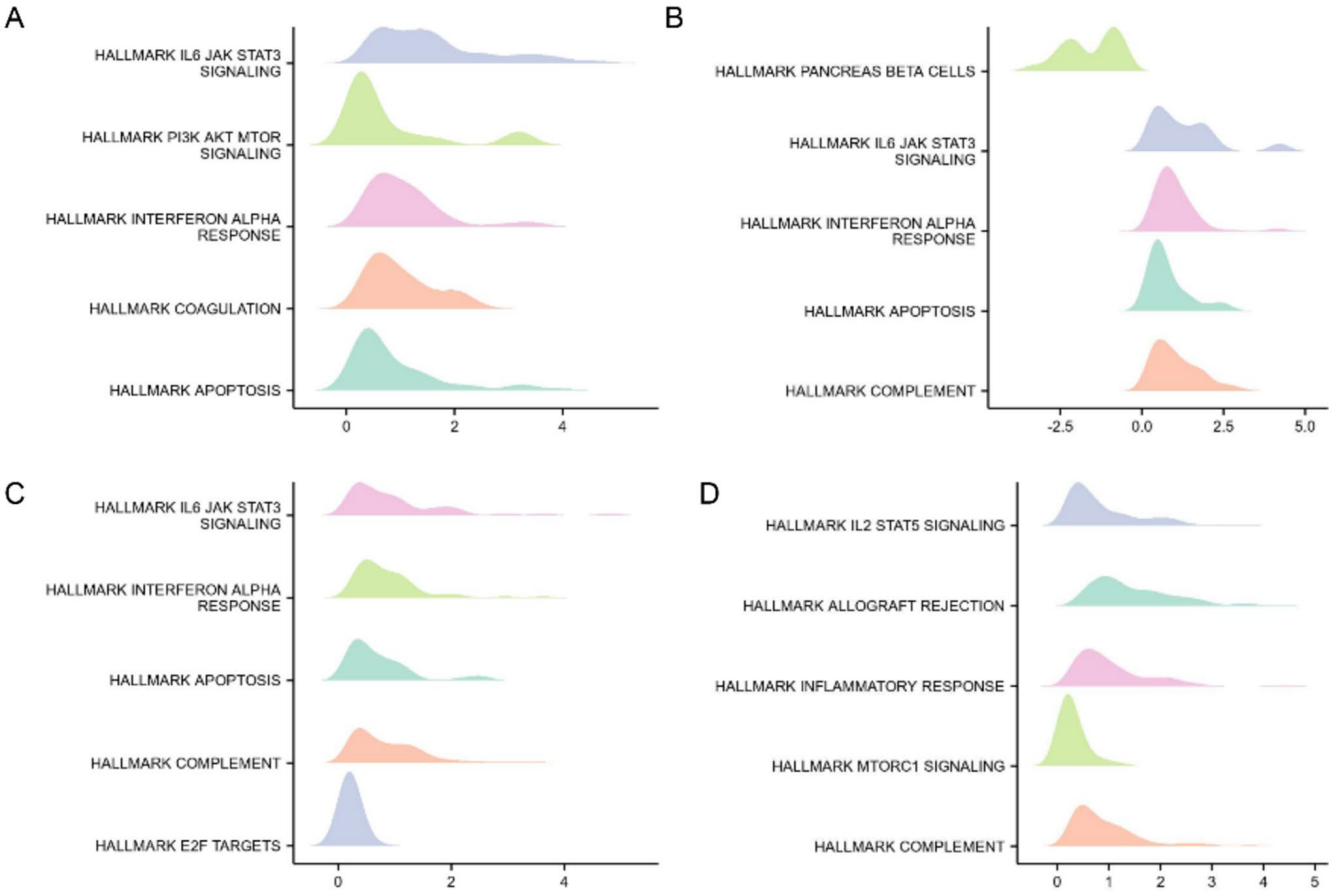
Top five signaling pathways associated with CXCL13 expression according to KEGG analyses for **A** BRCA, **B** CESC, **C** OV, and **D** UCEC

To deeply examine the functional roles of CXCL13, we performed GSEA and KEGG analysis for Pan-Gyn. GSEA results indicated that CXCL13 was involved in multiple pathways, such as interferon α pathway, apoptotic signaling pathway, and IL2_STAT5 pathway (Fig. 4). Results presented above suggest functions and pathways are mainly related to apoptosis and immune functions. Overexpression of CXCL13 suggests immune activation and metastasis according to literature reports (Ren et al. 2022). Notably, apoptotic pathways were significantly enriched in all four tumors. These evidences indicate that CXCL13 may not only promote inflammatory response but also be a hallmark of apoptosis. Taken together, GSEA results suggest that CXCL13-high patients have a better ability to induce apoptosis and immune cell infiltrates, and have a better prognosis correspondingly.

### Relationship between CXCL13 and TIME

**Fig. 5:**
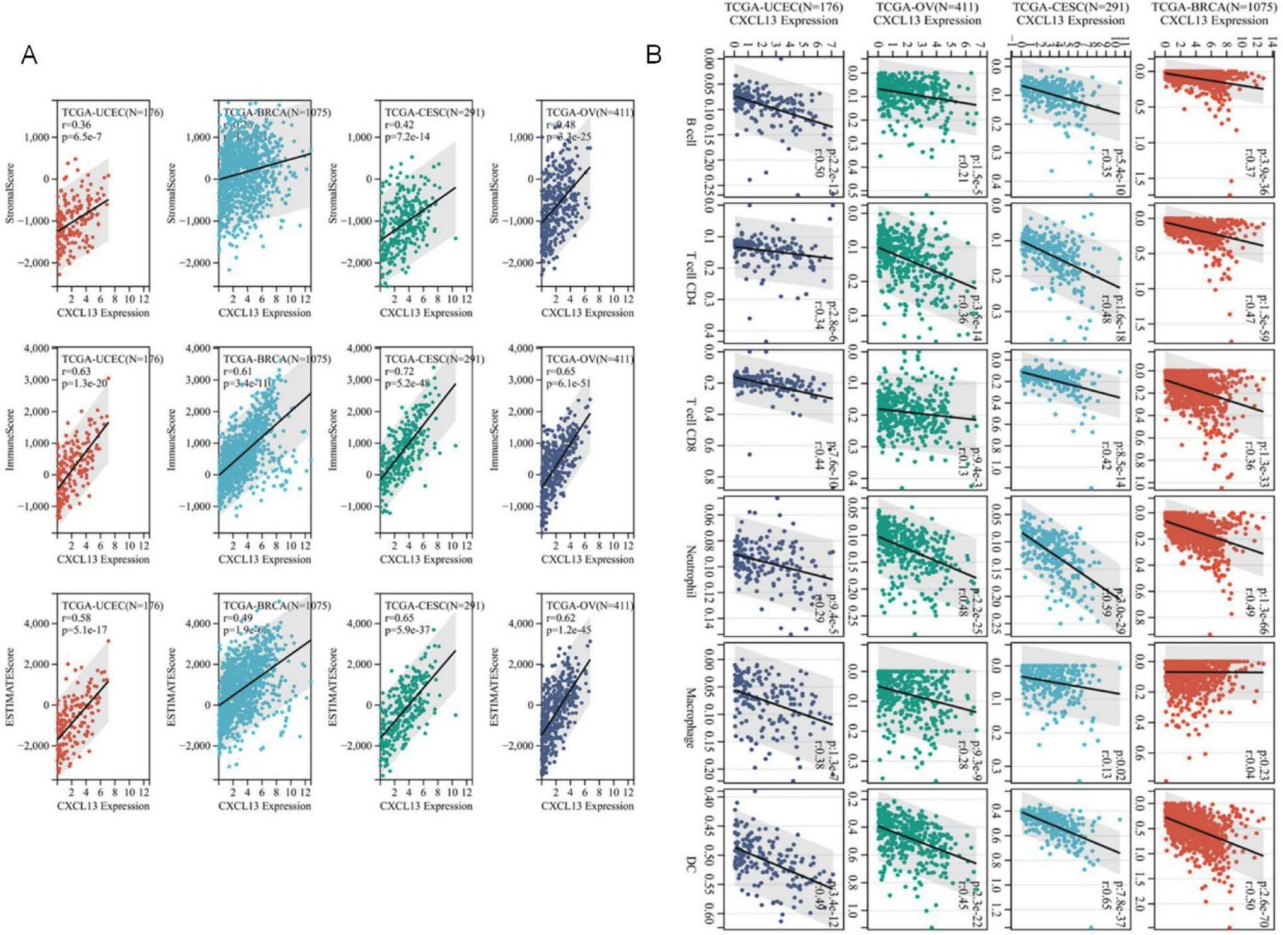
The CXCL13 expression correlated with immune infiltration. **A** Correlation analysis between CXCL13 expression levels and Immune/Stromal/Estimate Scores in Pan-Gyn; **B** The CXCL13 expression significantly correlated with the infiltration levels of six immune cell types in the TIMER database

Chemokine expression is essential for the regulation of immune cells (Ozga et al. 2021). Due to the clear connection between CXCL13 and the inflammatory response, we conducted a Pan-Gyn analysis of the association between the CXCL13 expression and the immune infiltration level based on multiple online tools. As shown in Fig. 5A, the expression of CXCL13 was positively associated with the Stromal Score and Immune Score (*R* > 0.6). We further used the TIMER online tool to examine the relationship between CXCL13 expression and immune cell subtypes. The results show that CXCL13 has a strong correlation (*R* > 0.4) with two immune infiltration cell types (neutrophil, dendritic), while a weak correlation with B cell, T cell, and macrophage (Fig. 5B). To further confirm this conclusion, xCell was used to assess the infiltration scores of 64 cell types in Pan-Gyn (Fig. S4A). In addition, we also assessed the Spearman correlation between the immune infiltration of 22 immune cells (CIBERSORT, LM22) and CXCL13 (Fig. S4B). The results showed that CXCL13 expression positively correlated with scores of most immune cells. To strengthen our conclusions, we further used algorithms of EPIC, MCPCOUNTER, and QUANTISEQ to validate the results, and the outcomes displayed a high degree of consistency (Fig. S4C–E). Together, those results indicated that the CXCL13-H group had significantly higher infiltration of multiple types of immune cells compared to the CXCL13-L group, which suggests that CXCL13-H is ‘hot’ tumor, whereas CXCL13-L is in a “cold” immune state (tumors with low immune infiltrate).

### Relationship between CXCL13 and TIME at the single-cell level

We explored the expression profiles of CXCL13, PDCD1, CTLA4, CD4, CD8A, and MA4A1 through a single‐cell RNA sequencing database in more depth. The results showed that the expression of CXCL13 was highly consistent with PDCD1, CD8A, and CTLA4 at the single-cell level, being enriched in depleted T cells, which suggests that CXCL13 is a stable biomarker of immunotherapy in Pan-Gyn (Figs. S5, S6). And CXCL13 was found to be mainly expressed in CD4^+^ and CD8^+^ T cells based on single‐cell RNA sequencing dataset. Then the relationship between the CXCL13 and apoptotic pathway was validated at the single-cell level. We found that stromal cells in the tumor microenvironment increase the expression of pro-apoptotic signals in all four tumors, such as fibroblasts. Using expression of CD3 and MS4A1 as surrogates of the presence of tertiary lymphoid structure (TLS), we revealed CXCL13 is a pivotal protein involved in the formation of TLSs. Single-cell sequencing analysis gives us an idea of CXCL13 in TIME. To this end, further studies will hopefully reveal its detailed mechanism.

### Construction and verification of the nomogram models

**Fig. 6:**
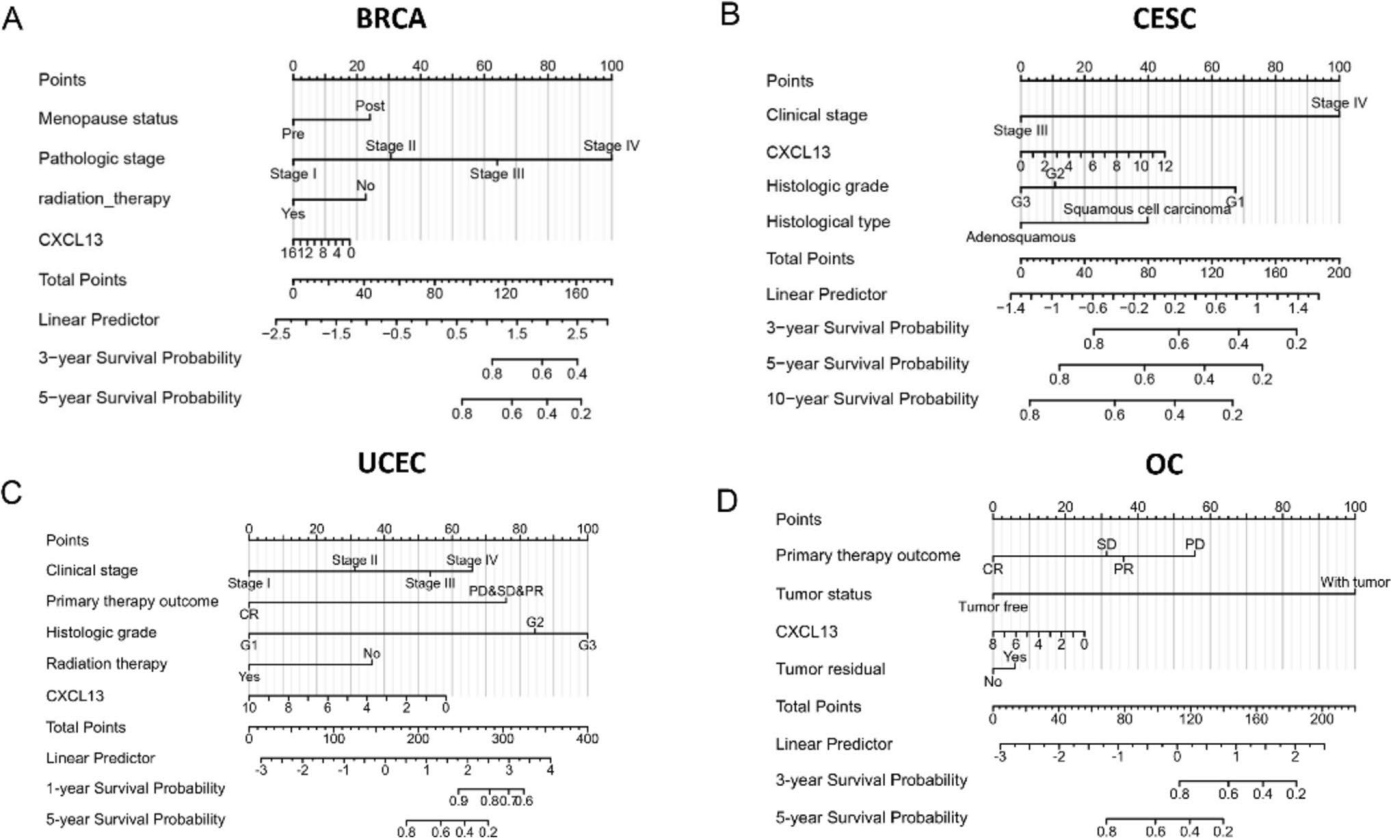
Nomograms predicting the 3- and 5-year overall survival probability of Pan-Gyn patients. **A** Nomogram of BRCA; **B** Nomogram of CESC; **C** Nomogram of OV; **D** Nomogram of UCEC

The role of CXCL13 in Pan-Gyn has been well established. However, the prognostic ability of CXCL13 alone is weak (Fig. S7A). CXCL13 and meaningful clinical variables in the multivariate Cox regression analysis were utilized in constructing the nomograms which have impact on predicting the OS of Pan-Gyn patients (Figs. S7, S8, S9). We conducted an internal verification and assessed the model’s discrimination and calibration. The evaluation of discrimination relied on the index of concordance (C-index) and the CI of all the models was over 0.7. In addition, calibration plots were utilized for the evaluation by examining the graph of predicted probabilities derived from the nomogram in comparison to the actual probabilities. The calibration curve displayed a strong agreement between the predicted and observed values of OS where the observed and predicted probabilities closely aligned with the 45-degree lines (Fig. S7B). These results indicated that the nomograms built with the amalgamation of features could predict the survival of Pan-Gyn patients more accurately (Fig. 6A–D).

## Discussion

Comprising stromal and tumor cells, the tumor microenvironment involves intricate cross-talk between these cells, which is orchestrated by an array of growth factors, cytokines, and chemokines (Hussain et al. 2019). Among the pivotal chemotactic factors, CXCL13 and CXCR5 assume critical roles in shaping the biology of cancer cells (Hussain et al. 2019). In this study, we systematically analyzed the relationship between CXCL13 and TIME in Pan-Gyn. Patients with high CXCL13 expression have a better prognosis, which is consistent in all four tumors. This result suggests that CXCL13 may exert an anti-tumor effect. The relationship between CXCL13 and immunity was further studied and CXCL13 was positively correlated with infiltrating immune cells, which means a close relationship between CXCL13 and anti-tumor immunity. CXCL13 strongly attracts B lymphocytes and plays an important role in regulating the spatial migration of secondary lymphocytes (Hsieh et al. 2022c). The Pan-Gyn impacts of CXCL13 have been relatively little studied. It has been confirmed that CXCL13 was contributed to TLSs formation in ovarian cancer (Ukita et al. 2022). This had also been proven in our research. Although there are few studies in cervical cancer, one study suggests that CXCL13 inhibits cervical cancer metastasis (Ma et al. 2020). In addition, there appears to be a link between CXCL13 and HPV virus (Liu et al. 2020; Varga et al. 2017). CXCL13 is involved in tumor immunity, though it exhibits duality in breast cancer (Razis et al. 2020). Moreover, the impact of CXCL13 on endometrial carcinoma is currently missing in the literature. Drawing from both the available literature and our own discoveries, it can be inferred that in Pan-Gyn, CXCL13 may potentially contribute to the initiation and progression of the disease through its influence on immune cell migration. However, this hypothesis needs further comprehensive investigation.

The nomogram, a prognostic tool, is widely used in clinical practice to provide individual patients with a single numerical estimate of event likelihood, such as mortality or recurrence, by condensing complex statistical predictive models (Iasonos et al. 2008). In our study, we find that CXCL13 alone is not sufficient to predict prognosis, but the situation may vary in other types of cancers, indicating extensive research prospects for CXCL13 (Liu et al. 2022; Magen et al. 2023). Upon further exploring, we performed univariate and multivariate Cox regression analyses and prognostic nomograms have been established using the ‘rms’ package subsequently. The concordance index and 95% confidence intervals were 0.763 (0.732–0.794) for the BRCA nomogram, 0.736 (0.684–0.788) for the CESC nomogram, 0.821 (0.793–0.849) for the UCEC nomogram, and 0.728 (0.707–0.749) for the OV nomogram. The nomogram exhibited good performance, as indicated by the calibration plots. The inclusion of CXCL13 in the prognostic model improved the predictive capacity and can offer a more precise individual prognosis for patients, providing crucial guidance for treatment and prognosis. Given its capability to effectively predict OS and its strong alignment with real incidence data, the model holds significant potential for clinical applications. Furthermore, CXCL13 is a secreted protein and can be detected in serum (Mukama et al. 2022; Peres et al. 2021). If the prognostic model can be constructed based on the level of serum, it will be more practical in clinical practice. Nevertheless, several limitations should also be noted. First, external validation sets and experimental validation are missing. Second, while our nomograms underwent internal validation via bootstrap validation, future research is warranted for the external validation of these nomograms. Therefore, more patients who received long-term follow-up need constant attention to improve nomogram model.

There are a few things of interest to note here. Using GSEA analysis, we found that patients with high CXCL13 expression were enriched in the apoptotic pathway in all four tumors. The apoptotic pathway and CXCL13 have been reported only in a few diseases such as breast cancer, lung adenocarcinoma, and cerebrovascular disease (Rayasam et al. 2022; Smedbakken et al. 2012; Tian et al. 2021). CXCL13 promotes MDA-MB-231 cell apoptosis by CXCL13-CXCR5-ERK signaling pathway in breast cancer (Ma et al. 2018). The results showed that the CXCL13 may also inhibit tumor progression and improve the prognosis of patients by inducing apoptosis of tumor cells. Beyond these, the single-cell sequencing results in Pan-Gyn suggest apoptosis also occurs in stromal cells which is likely to profoundly affect the tumor progression through effects on the microenvironment. Conversely, a few studies had reached the opposite conclusions. For instance, overexpression of CXCL13 rescued apoptosis in myasthenia gravis (Wu et al. 2019). Apart from this, CXCL13 may also directly modulate some tumors’ growth by preventing their apoptosis (Hussain et al. 2021). Its double-sided role in apoptosis remains to be explored and the mechanisms remain ambiguous. Furthermore, it is essential to acknowledge that CXCL13 might facilitate the onset, progression, and adverse prognosis of cancer by triggering additional oncogenic pathways, like the JAK-STAT pathway, via the chemokine pathway or cytokine receptor pathway (Zhang et al. 2022). This may be related to the duality of the immune system in tumor progression (Ozga et al. 2021). In a nutshell, CXCL13 might play a role in promoting malignant behaviors such as migration, invasion, and apoptosis through the aforementioned signaling pathway.

## Conclusion

Briefly, this study is a thorough analysis of CXCL13 genes and mechanism in Pan-Gyn. Then prognostic nomograms were constructed. The nomogram created with CXCL13 provides a more precise prediction, which can be of greater assistance to clinicians in formulating clinical decisions and tailoring treatment plans. Herein, the detailed mechanism of CXCL13 in pan-gynecological tumors deserves further elucidation.

### Supplementary Information

Below is the link to the electronic supplementary material.

**Appendix A. Supplementary**: **Fig. S1** Top 15 most-highly mutated genes between two groups in BRCA (**A**) and CESC (**B**). **Fig. S2** Top 15 most-highly mutated genes between two groups in UCEC (**A**) and OV (**B**). **Fig. S3** **A**: The protein expression of CXCL13 in immunohistochemical images; **B**–**C**: CXCL13 expression was associated with significantly more unfavorable DSS and PFI (clockwise direction: BRCA; CESC; OV; UCEC); **D**: Relationship between CXCL13 and tumor stemness (left: mRNAsi; right: mDNAsi). *p < 0.05, **p < 0.01, ***p < 0.001. **Fig. S4** Relationship between CXCL13 levels and immune infiltrates analyzed **A**: xCell; **B**: C IBERSORT; **C**: EPIC; **D**: QUANTISEQ; E: MCPcounter. *p < 0.05, **p < 0.01, ***p < 0.001. **Fig. S5** Single-cell sequencing analyzing CXCL13 co-expression and GSEA (apoptosis pathway) analyses for BRCA (**A**) and CESC (**B**). **Fig. S6** Single-cell sequencing analyzing CXCL13 co-expression and GSEA (apoptosis pathway) analyses for UCEC (A) and OV (B). **Fig. S7** **A**: ROC curves indicating that CXCL13 could predict OS; **B**: Calibration curves of nomograms; **C**: Survival analysis of CXCL13 in GEO cohorts (left to right: BRCA; OV; UCEC). *p < 0.05, **p < 0.01, ***p < 0.001. **Fig. S8** Forest plot of multivariate COX regression analysis for BRCA (**A**) and CESC (**B**). **Fig. S9** Forest plot of multivariate COX regression analysis for UCEC (**A**) and OV (**B**). (PDF 4019 KB)

## Data Availability

These data were derived from the following resources available in the public domain (list in “Materials and methods”). Further details and other data that support the findings of this study are available from the corresponding author upon request.

## References

[CR1] Berger AC, Korkut A, Kanchi RS, Hegde AM, Lenoir W, Liu W, Liu Y, Fan H, Shen H, Ravikumar V, Rao A, Schultz A, Li X, Sumazin P, Williams C, Mestdagh P, Gunaratne PH, Yau C, Bowlby R, Robertson AG, Tiezzi DG, Wang C, Cherniack AD, Godwin AK, Kuderer NM, Rader JS, Zuna RE, Sood AK, Lazar AJ, Ojesina AI, Adebamowo C, Adebamowo SN, Baggerly KA, Chen TW, Chiu HS, Lefever S, Liu L, MacKenzie K, Orsulic S, Roszik J, Shelley CS, Song Q, Vellano CP, Wentzensen N, Weinstein JN, Mills GB, Levine DA, Akbani R (2018). A comprehensive pan-cancer molecular study of gynecologic and breast cancers. Cancer Cell.

[CR2] Cannistra SA, Pujade-Lauraine E (2019). Progress and promise in treating gynecologic cancers. J Clin Oncol.

[CR3] Chung SH, Woldenberg N, Roth AR, Masamed R, Conlon W, Cohen JG, Joines MM, Patel MK (2020). BRCA and beyond: comprehensive image-rich review of hereditary breast and gynecologic cancer syndromes. Radiographics.

[CR4] Cosgrove J, Novkovic M, Albrecht S, Pikor NB, Zhou Z, Onder L, Morbe U, Cupovic J, Miller H, Alden K, Thuery A, O'Toole P, Pinter R, Jarrett S, Taylor E, Venetz D, Heller M, Uguccioni M, Legler DF, Lacey CJ, Coatesworth A, Polak WG, Cupedo T, Manoury B, Thelen M, Stein JV, Wolf M, Leake MC, Timmis J, Ludewig B, Coles MC (2020). B cell zone reticular cell microenvironments shape CXCL13 gradient formation. Nat Commun.

[CR5] Harris BS, Bishop KC, Kuller JA, Ford AC, Muasher LC, Cantrell SE, Price TM (2020). Hormonal management of menopausal symptoms in women with a history of gynecologic malignancy. Menopause.

[CR6] Hsieh CH, Jian CZ, Lin LI, Low GS, Ou PY, Hsu C, Ou DL (2022). Potential role of CXCL13/CXCR5 signaling in immune checkpoint inhibitor treatment in cancer. Cancers (basel).

[CR7] Hsieh CH, Jian CZ, Lin LI, Low GS, Ou PY, Hsu C, Ou DL (2022). Potential role of CXCL13/CXCR5 signaling in immune checkpoint inhibitor treatment in cancer. Cancers (basel).

[CR8] Hsieh CH, Jian CZ, Lin LI, Low GS, Ou PY, Hsu C, Ou DL (2022). Potential role of CXCL13/CXCR5 signaling in immune checkpoint inhibitor treatment in cancer. Cancers (basel).

[CR9] Husby A, Wohlfahrt J, Oyen N, Melbye M (2018). Pregnancy duration and breast cancer risk. Nat Commun.

[CR10] Hussain M, Adah D, Tariq M, Lu Y, Zhang J, Liu J (2019). CXCL13/CXCR5 signaling axis in cancer. Life Sci.

[CR11] Hussain M, Liu J, Wang GZ, Zhou GB (2021). CXCL13 signaling in the tumor microenvironment. Adv Exp Med Biol.

[CR12] Iasonos A, Schrag D, Raj GV, Panageas KS (2008). How to build and interpret a nomogram for cancer prognosis. J Clin Oncol.

[CR13] Ilango S, Paital B, Jayachandran P, Padma PR, Nirmaladevi R (2020). Epigenetic alterations in cancer. Front Biosci (landmark Ed).

[CR14] Kalaitzopoulos DR, Mitsopoulou A, Iliopoulou SM, Daniilidis A, Samartzis EP, Economopoulos KP (2020). Association between endometriosis and gynecological cancers: a critical review of the literature. Arch Gynecol Obstet.

[CR15] Lee JM, Peer CJ, Yu M, Amable L, Gordon N, Annunziata CM, Houston N, Goey AK, Sissung TM, Parker B, Minasian L, Chiou VL, Murphy RF, Widemann BC, Figg WD, Kohn EC (2017). Sequence-specific pharmacokinetic and pharmacodynamic phase I/Ib study of olaparib tablets and carboplatin in women's cancer. Clin Cancer Res.

[CR16] Li T, Fu J, Zeng Z, Cohen D, Li J, Chen Q, Li B, Liu XS (2020). TIMER2.0 for analysis of tumor-infiltrating immune cells. Nucleic Acids Res.

[CR17] Liu YH, Chen HL, Xu BQ, Wei K, Ying XY (2020). A preliminary study on the immune responses of HPV16-E7 by combined intranasal immunization with lymphotoxin. Ginekol Pol.

[CR18] Liu B, Zhang Y, Wang D, Hu X, Zhang Z (2022). Single-cell meta-analyses reveal responses of tumor-reactive CXCL13(+) T cells to immune-checkpoint blockade. Nat Cancer.

[CR19] Ma JJ, Jiang L, Tong DY, Ren YN, Sheng MF, Liu HC (2018). CXCL13 inhibition induce the apoptosis of MDA-MB-231 breast cancer cells through blocking CXCR5/ERK signaling pathway. Eur Rev Med Pharmacol Sci.

[CR20] Ma D, Fan SB, Hua N, Li GH, Chang Q, Liu X (2020). Hypermethylation of single CpG dinucleotides at the promoter of CXCL13 gene promoting cell migration in cervical cancer. Curr Cancer Drug Targets.

[CR21] Magen A, Hamon P, Fiaschi N, Soong BY, Park MD, Mattiuz R, Humblin E, Troncoso L, D'Souza D, Dawson T, Kim J, Hamel S, Buckup M, Chang C, Tabachnikova A, Schwartz H, Malissen N, Lavin Y, Soares-Schanoski A, Giotti B, Hegde S, Ioannou G, Gonzalez-Kozlova E, Hennequin C, Le Berichel J, Zhao Z, Ward SC, Fiel I, Kou B, Dobosz M, Li L, Adler C, Ni M, Wei Y, Wang W, Atwal GS, Kundu K, Cygan KJ, Tsankov AM, Rahman A, Price C, Fernandez N, He J, Gupta NT, Kim-Schulze S, Gnjatic S, Kenigsberg E, Deering RP, Schwartz M, Marron TU, Thurston G, Kamphorst AO, Merad M (2023). Intratumoral dendritic cell-CD4(+) T helper cell niches enable CD8(+) T cell differentiation following PD-1 blockade in hepatocellular carcinoma. Nat Med.

[CR22] Martinez X, Chavent M, Baaden M (2020). Visualizing protein structures - tools and trends. Biochem Soc Trans.

[CR23] Monneau YR, Luo L, Sankaranarayanan NV, Nagarajan B, Vives RR, Baleux F, Desai UR, Arenzana-Seidedos F, Lortat-Jacob H (2017). Solution structure of CXCL13 and heparan sulfate binding show that GAG binding site and cellular signalling rely on distinct domains. Open Biol.

[CR24] Morad G, Helmink BA, Sharma P, Wargo JA (2021). Hallmarks of response, resistance, and toxicity to immune checkpoint blockade. Cell.

[CR25] Mukama T, Fortner RT, Katzke V, Hynes LC, Petrera A, Hauck SM, Johnson T, Schulze M, Schiborn C, Rostgaard-Hansen AL, Tjonneland A, Overvad K, Perez M, Crous-Bou M, Chirlaque MD, Amiano P, Ardanaz E, Watts EL, Travis RC, Sacerdote C, Grioni S, Masala G, Signoriello S, Tumino R, Gram IT, Sandanger TM, Sartor H, Lundin E, Idahl A, Heath AK, Dossus L, Weiderpass E, Kaaks R (2022). Prospective evaluation of 92 serum protein biomarkers for early detection of ovarian cancer. Br J Cancer.

[CR26] Newman AM, Liu CL, Green MR, Gentles AJ, Feng W, Xu Y, Hoang CD, Diehn M, Alizadeh AA (2015). Robust enumeration of cell subsets from tissue expression profiles. Nat Methods.

[CR27] Ozga AJ, Chow MT, Luster AD (2021). Chemokines and the immune response to cancer. Immunity.

[CR28] Pan Z, Zhu T, Liu Y, Zhang N (2022). Role of the CXCL13/CXCR5 axis in autoimmune diseases. Front Immunol.

[CR29] Peres LC, Townsend MK, Birmann BM, Conejo-Garcia JR, Kim Y, Kubzansky LD, Magpantay LI, Martinez-Maza O, Tworoger SS (2021). Circulating biomarkers of inflammation and ovarian cancer risk in the nurses' health studies. Cancer Epidemiol Biomarkers Prev.

[CR30] Pham MM, Ngoi N, Peng G, Tan D, Yap TA (2021). Development of poly(ADP-ribose) polymerase inhibitor and immunotherapy combinations: progress, pitfalls, and promises. Trends Cancer.

[CR31] Racle J, de Jonge K, Baumgaertner P, Speiser DE, Gfeller D (2017). Simultaneous enumeration of cancer and immune cell types from bulk tumor gene expression data. Elife.

[CR32] Rayasam A, Kijak JA, Kissel L, Choi YH, Kim T, Hsu M, Joshi D, Laaker CJ, Cismaru P, Lindstedt A, Kovacs K, Vemuganti R, Chiu SY, Priyathilaka TT, Sandor M, Fabry Z (2022). CXCL13 expressed on inflamed cerebral blood vessels recruit IL-21 producing TFH cells to damage neurons following stroke. J Neuroinflammation.

[CR33] Razis E, Kalogeras KT, Kotsantis I, Koliou GA, Manousou K, Wirtz R, Veltrup E, Patsea H, Poulakaki N, Dionysopoulos D, Pervana S, Gogas H, Koutras A, Pentheroudakis G, Christodoulou C, Linardou H, Pavlakis K, Koletsa T, Pectasides D, Zagouri F, Fountzilas G (2020). The role of CXCL13 and CXCL9 in early breast cancer. Clin Breast Cancer.

[CR34] Ren J, Lan T, Liu T, Liu Y, Shao B, Men K, Ma Y, Liang X, Wei YQ, Luo M, Wei XW (2022). CXCL13 as a novel immune checkpoint for regulatory B cells and its role in tumor metastasis. J Immunol.

[CR35] Ritchie G, Gasper H, Man J, Lord S, Marschner I, Friedlander M, Lee CK (2018). Defining the most appropriate primary end point in phase 2 trials of immune checkpoint inhibitors for advanced solid cancers: a systematic review and meta-analysis. JAMA Oncol.

[CR36] Rosenberg EJ, Herrington J, Rajasekaran D, Murphy JW, Pantouris G, Lolis EJ (2020). The N-terminal length and side-chain composition of CXCL13 affect crystallization, structure and functional activity. Acta Crystallogr D Struct Biol.

[CR37] Rubio AJ, Porter T, Zhong X (2020). Duality of B cell-CXCL13 axis in tumor immunology. Front Immunol.

[CR38] Singh M, Kumar V, Sehrawat N, Yadav M, Chaudhary M, Upadhyay SK, Kumar S, Sharma V, Kumar S, Dilbaghi N, Sharma AK (2022). Current paradigms in epigenetic anticancer therapeutics and future challenges. Semin Cancer Biol.

[CR39] Smedbakken LM, Halvorsen B, Daissormont I, Ranheim T, Michelsen AE, Skjelland M, Sagen EL, Folkersen L, Krohg-Sorensen K, Russell D, Holm S, Ueland T, Fevang B, Hedin U, Yndestad A, Gullestad L, Hansson GK, Biessen EA, Aukrust P (2012). Increased levels of the homeostatic chemokine CXCL13 in human atherosclerosis—potential role in plaque stabilization. Atherosclerosis.

[CR40] Sun D, Wang J, Han Y, Dong X, Ge J, Zheng R, Shi X, Wang B, Li Z, Ren P, Sun L, Yan Y, Zhang P, Zhang F, Li T, Wang C (2021). TISCH: a comprehensive web resource enabling interactive single-cell transcriptome visualization of tumor microenvironment. Nucleic Acids Res.

[CR41] Tian C, Li C, Zeng Y, Liang J, Yang Q, Gu F, Hu Y, Liu L (2021). Identification of CXCL13/CXCR5 axis's crucial and complex effect in human lung adenocarcinoma. Int Immunopharmacol.

[CR42] Trayes KP, Cokenakes S (2021). Breast cancer treatment. Am Fam Physician.

[CR43] Ukita M, Hamanishi J, Yoshitomi H, Yamanoi K, Takamatsu S, Ueda A, Suzuki H, Hosoe Y, Furutake Y, Taki M, Abiko K, Yamaguchi K, Nakai H, Baba T, Matsumura N, Yoshizawa A, Ueno H, Mandai M (2022). CXCL13-producing CD4+ T cells accumulate in the early phase of tertiary lymphoid structures in ovarian cancer. JCI Insight.

[CR44] Vanacker H, Harter P, Labidi-Galy SI, Banerjee S, Oaknin A, Lorusso D, Ray-Coquard I (2021). PARP-inhibitors in epithelial ovarian cancer: actual positioning and future expectations. Cancer Treat Rev.

[CR45] Varga N, Mozes J, Keegan H, White C, Kelly L, Pilkington L, Benczik M, Zsuzsanna S, Sobel G, Koiss R, Babarczi E, Nyiri M, Kovacs L, Attila S, Kaltenecker B, Geresi A, Kocsis A, O'Leary J, Martin CM, Jeney C (2017). The value of a novel panel of cervical cancer biomarkers for triage of HPV positive patients and for detecting disease progression. Pathol Oncol Res.

[CR46] Verma N, Vinik Y, Saroha A, Nair NU, Ruppin E, Mills G, Karn T, Dubey V, Khera L, Raj H, Maina F, Lev S (2020). Synthetic lethal combination targeting BET uncovered intrinsic susceptibility of TNBC to ferroptosis. Sci Adv.

[CR47] Wagener-Ryczek S, Merkelbach-Bruse S, Siemanowski J (2021). Biomarkers for homologous recombination deficiency in cancer. J Pers Med.

[CR48] Wu DM, Wen X, Han XR, Wang S, Wang YJ, Shen M, Fan SH, Zhuang J, Zhang ZF, Shan Q, Li MQ, Hu B, Sun CH, Lu J, Zheng YL (2019). Micro-RNA-143 inhibits proliferation and promotes apoptosis of thymocytes by targeting CXCL13 in a myasthenia gravis mouse model. Am J Physiol Cell Physiol.

[CR49] Xiong J, Yan L, Zou C, Wang K, Chen M, Xu B, Zhou Z, Zhang D (2021). Integrins regulate stemness in solid tumor: an emerging therapeutic target. J Hematol Oncol.

[CR50] Xu T, Yu S, Zhang J, Wu S (2021). Dysregulated tumor-associated macrophages in carcinogenesis, progression and targeted therapy of gynecological and breast cancers. J Hematol Oncol.

[CR51] Yang M, Lu J, Zhang G, Wang Y, He M, Xu Q, Xu C, Liu H (2021). CXCL13 shapes immunoactive tumor microenvironment and enhances the efficacy of PD-1 checkpoint blockade in high-grade serous ovarian cancer. J Immunother Cancer.

[CR52] Yin X, Kong L, Liu P (2021). Identification of prognosis-related molecular subgroups based on DNA methylation in pancreatic cancer. Clin Epigenetics.

[CR53] Zhang H, Yin H, Chen J, Yuan Y (2022). An integrated pan-cancer analysis of 33 human cancers reveals the potential clinical implications and immunotherapeutic value of C-X-C Motif Chemokine Ligand 13. Front Oncol.

[CR54] Zhu H, Gu X, Xia L, Zhou Y, Bouamar H, Yang J, Ding X, Zwieb C, Zhang J, Hinck AP, Sun LZ, Zhu X (2018). A novel TGFβ trap blocks chemotherapeutics-induced TGFβ1 signaling and enhances their anticancer activity in gynecologic cancers. Clin Cancer Res.

