# Supplementary figures and images for: Transcriptome analysis reveals the clinical significance of CXCL13 in Pan-Gyn tumors
Source: J Cancer Res Clin Oncol. 2024 Mar 9;150(3):116. doi: 10.1007/s00432-024-05619-3 (PMC10923744; doi:10.1007/s00432-024-05619-3)

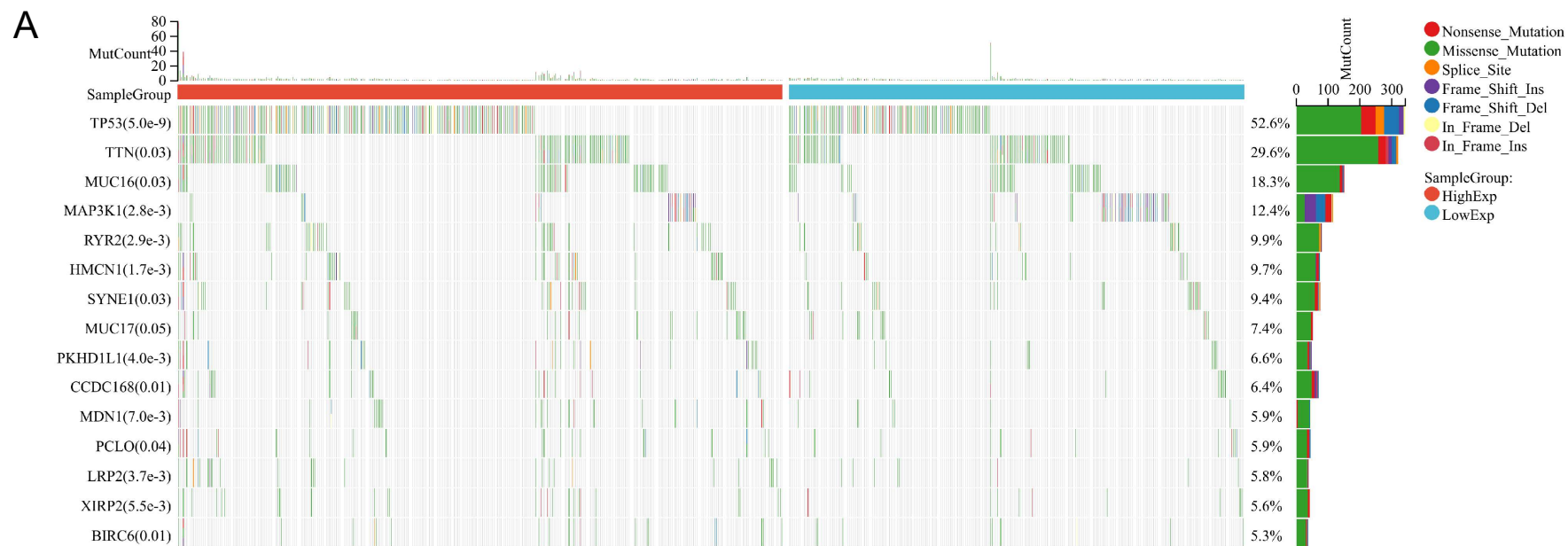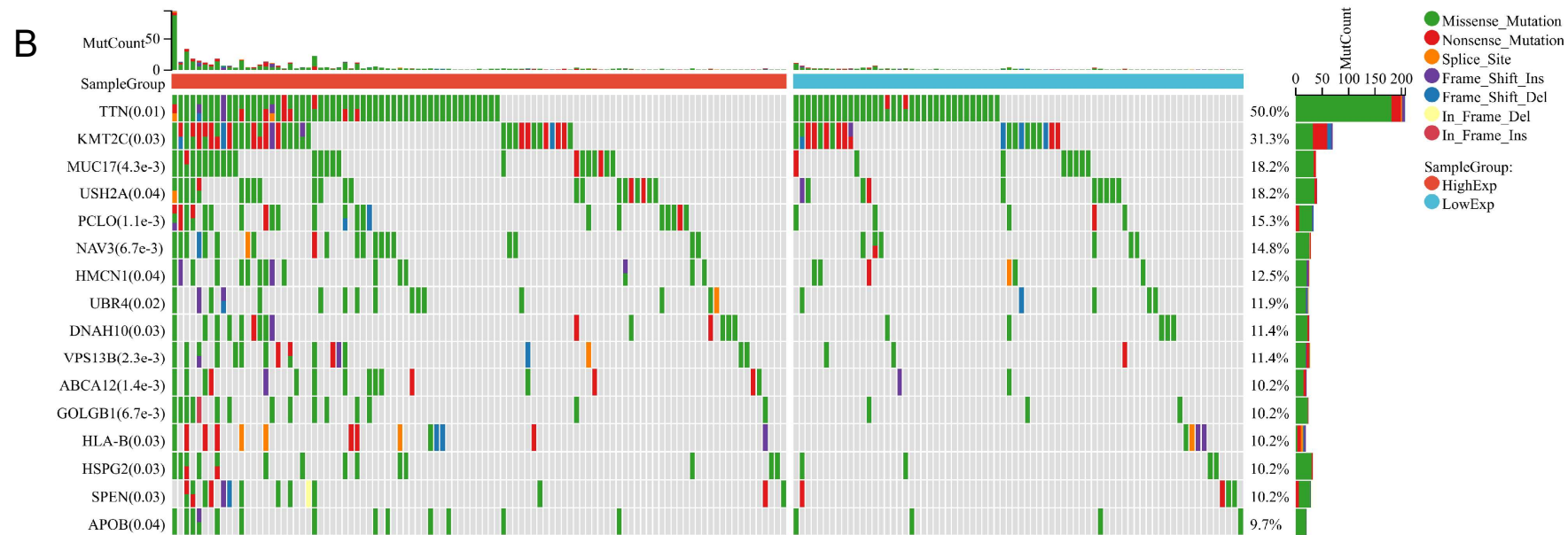

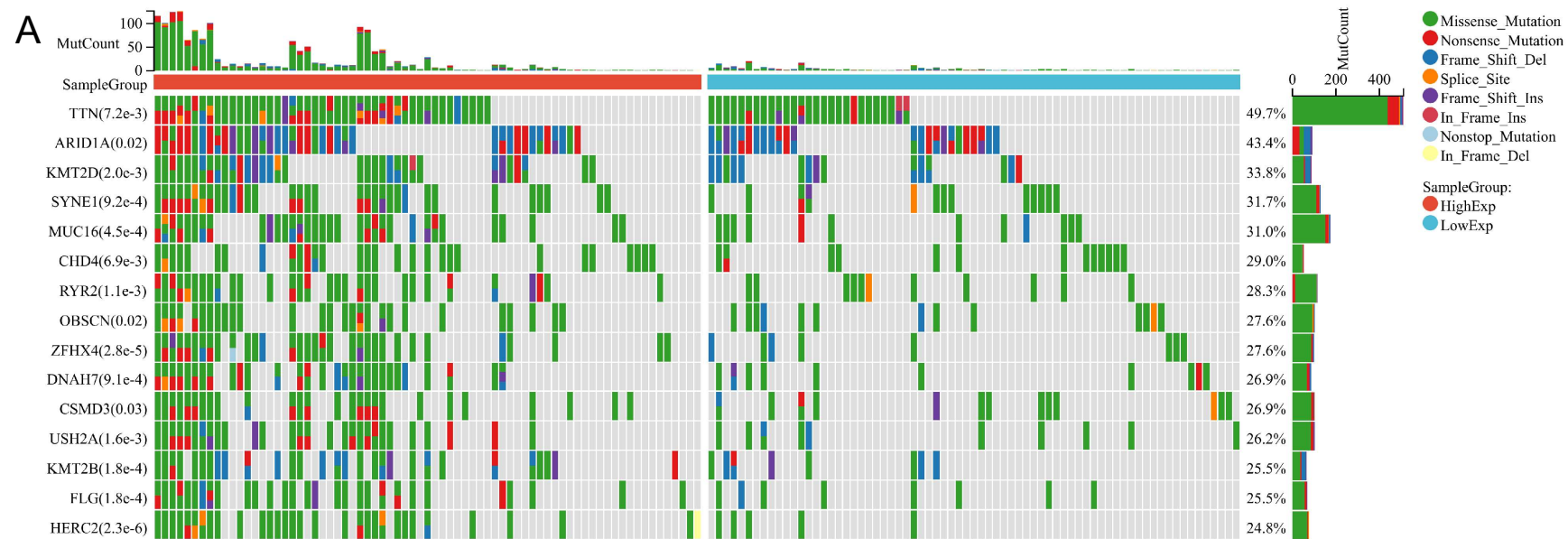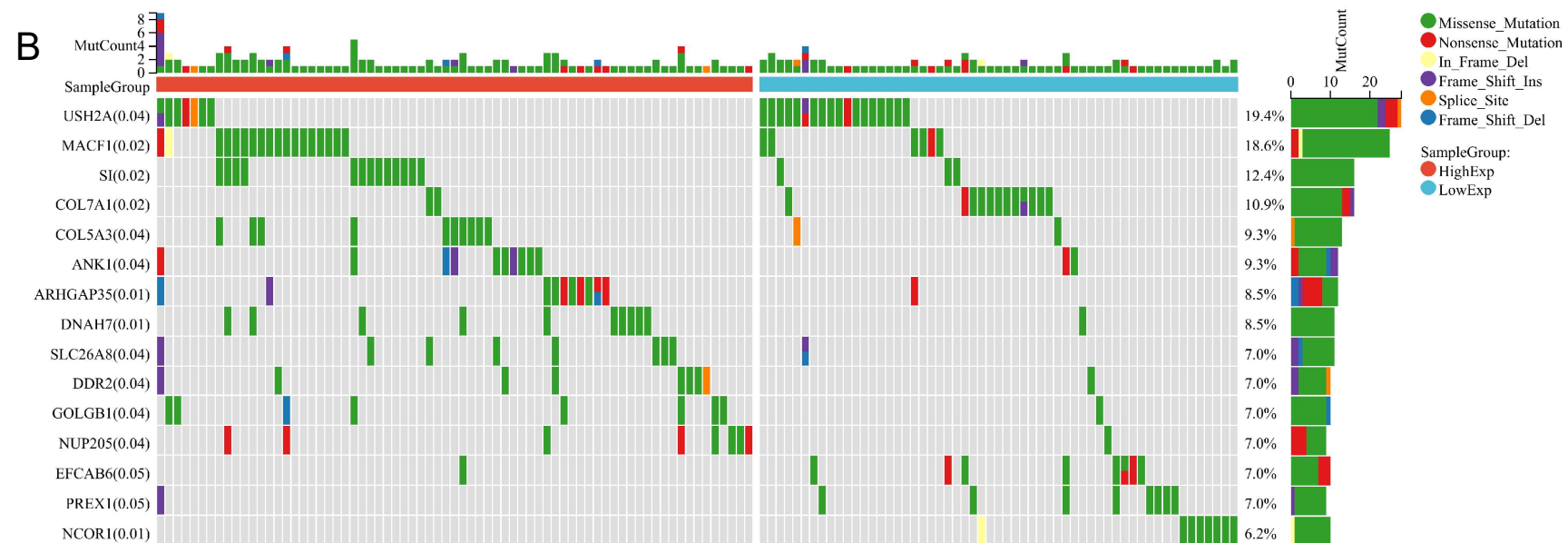

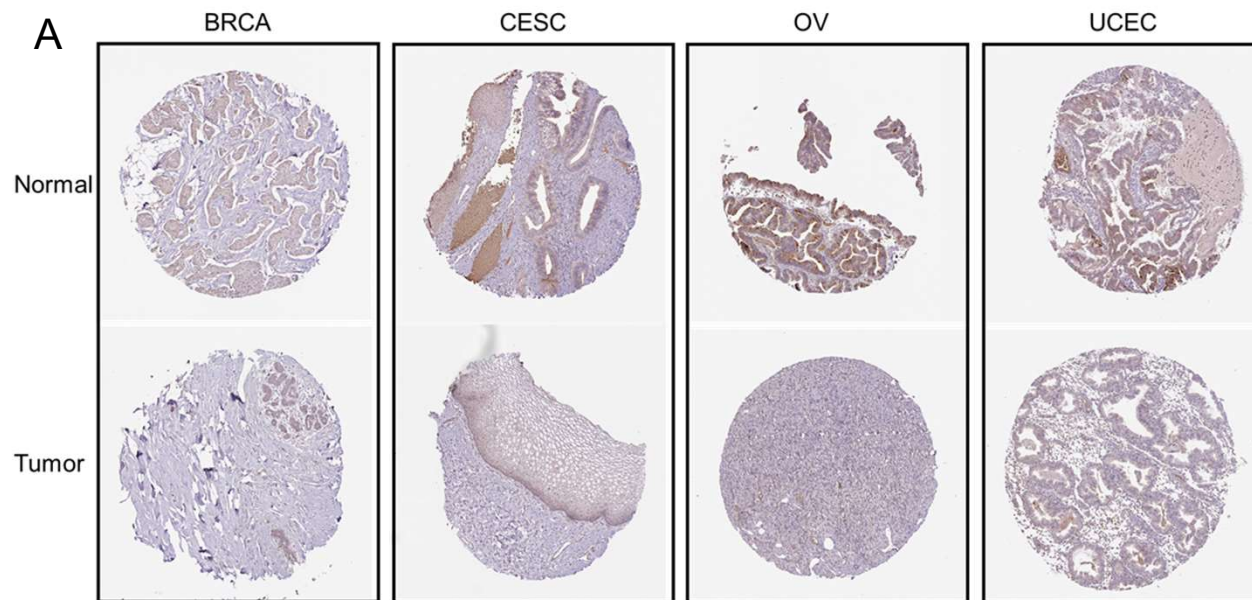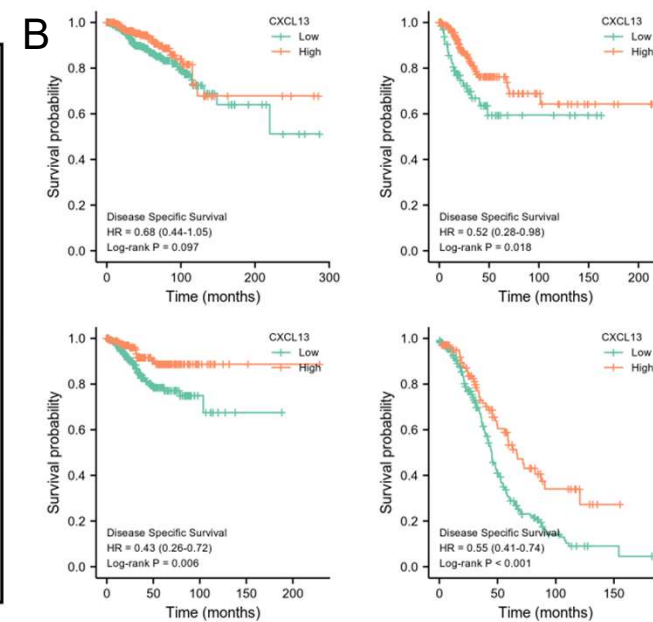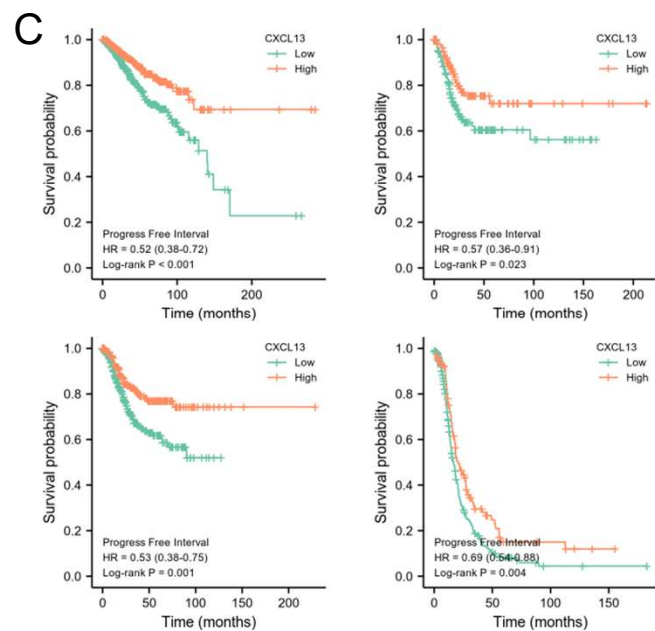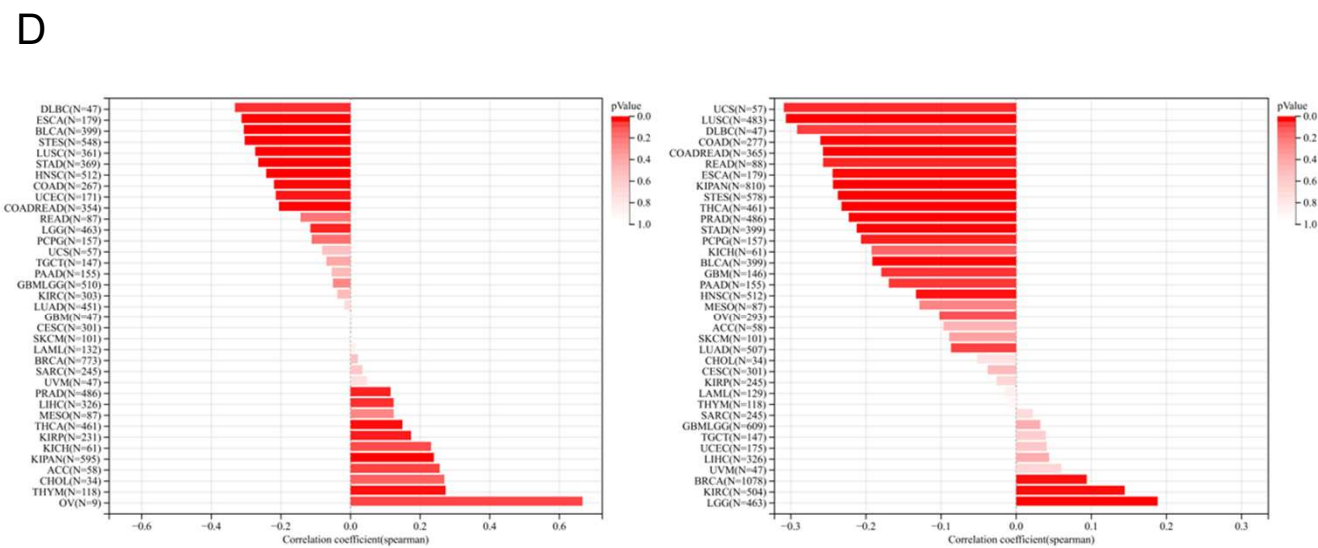



# A BRCA\_GSE110686

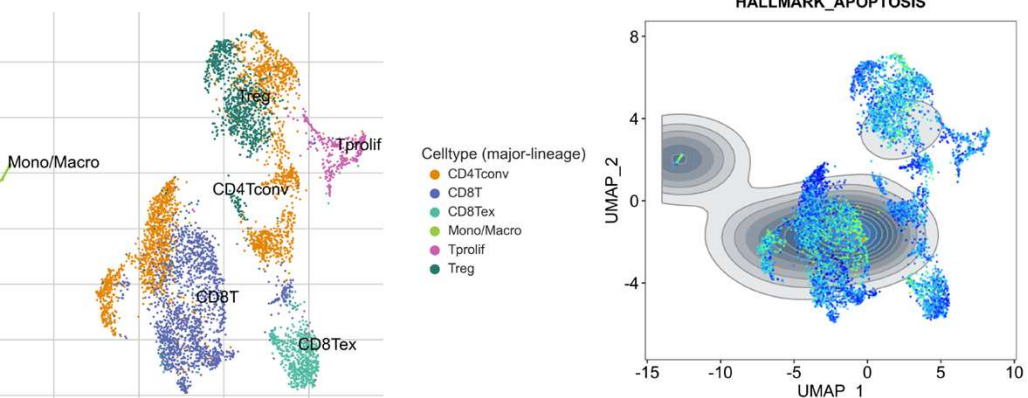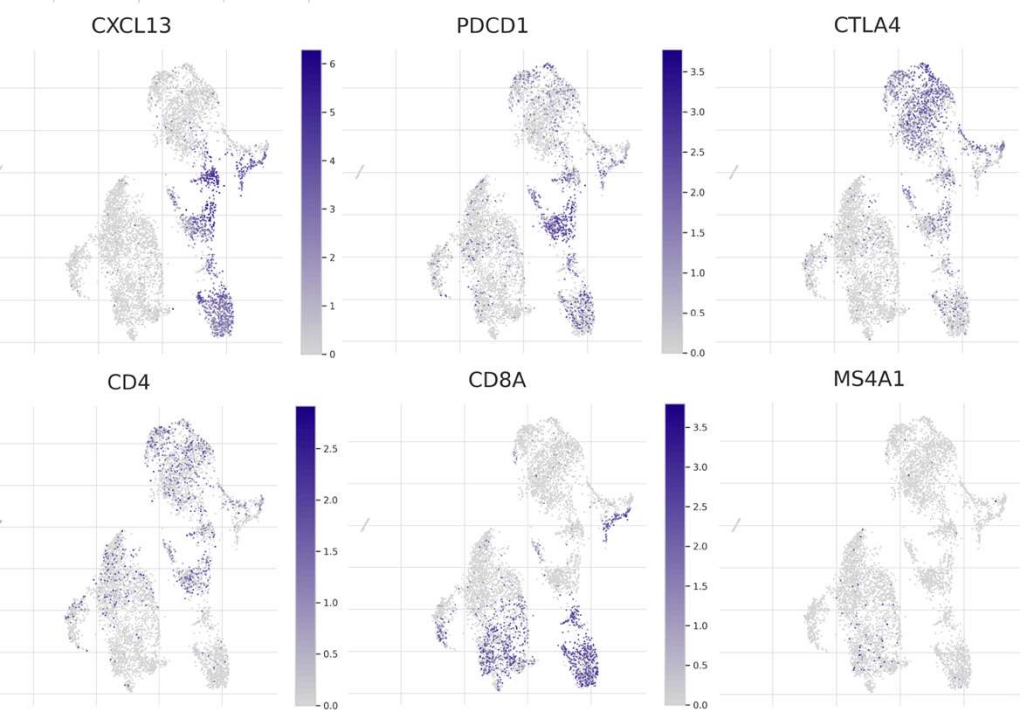

# B CESC\_GSE168652

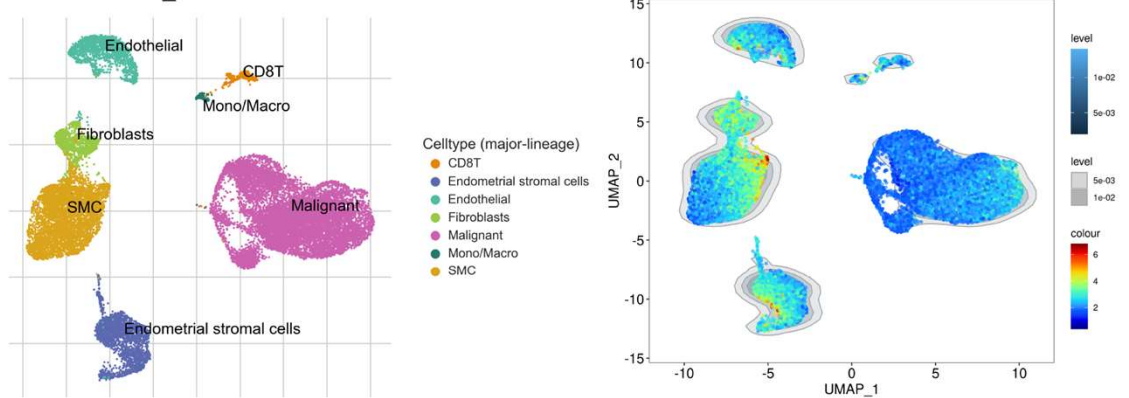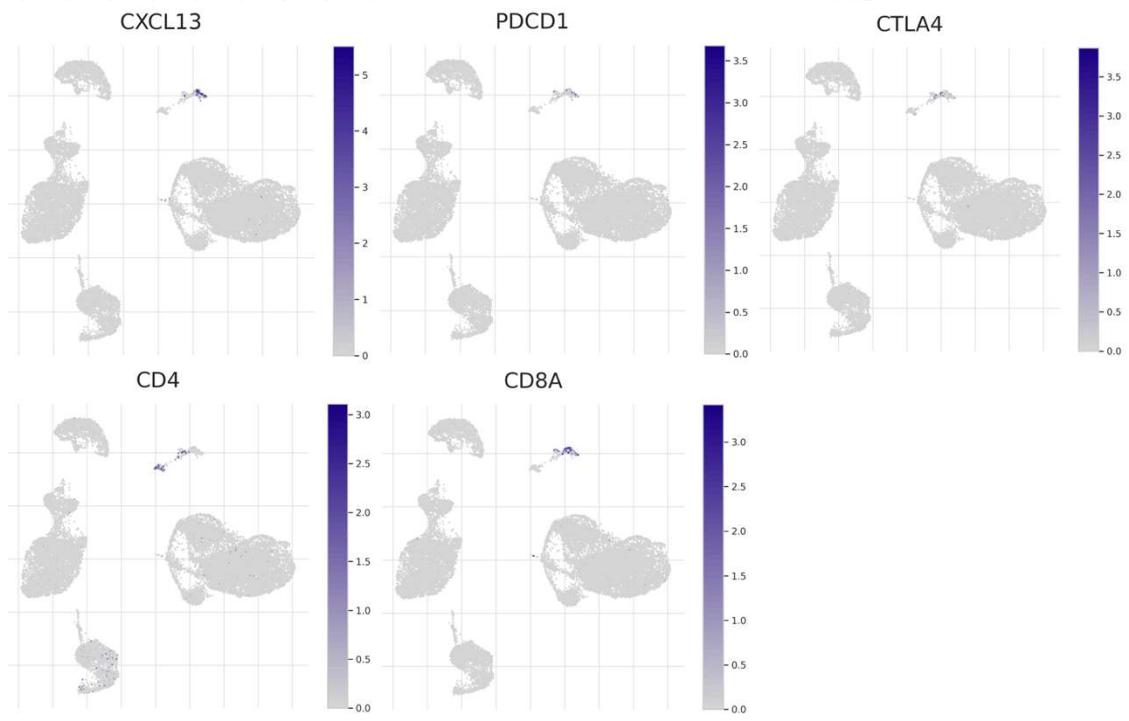

A

UCEC\_GSE139555

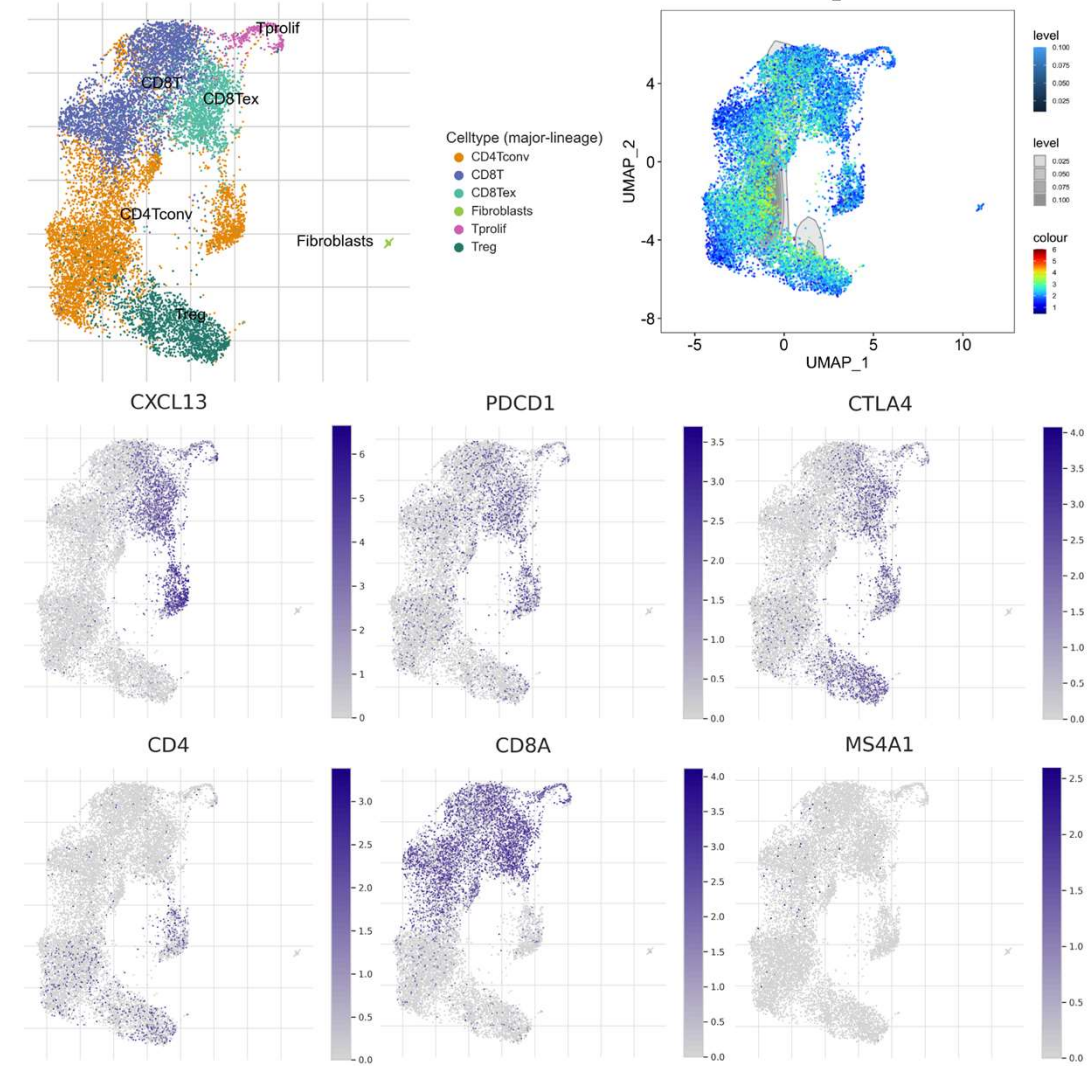

B

OV\_GSE154600

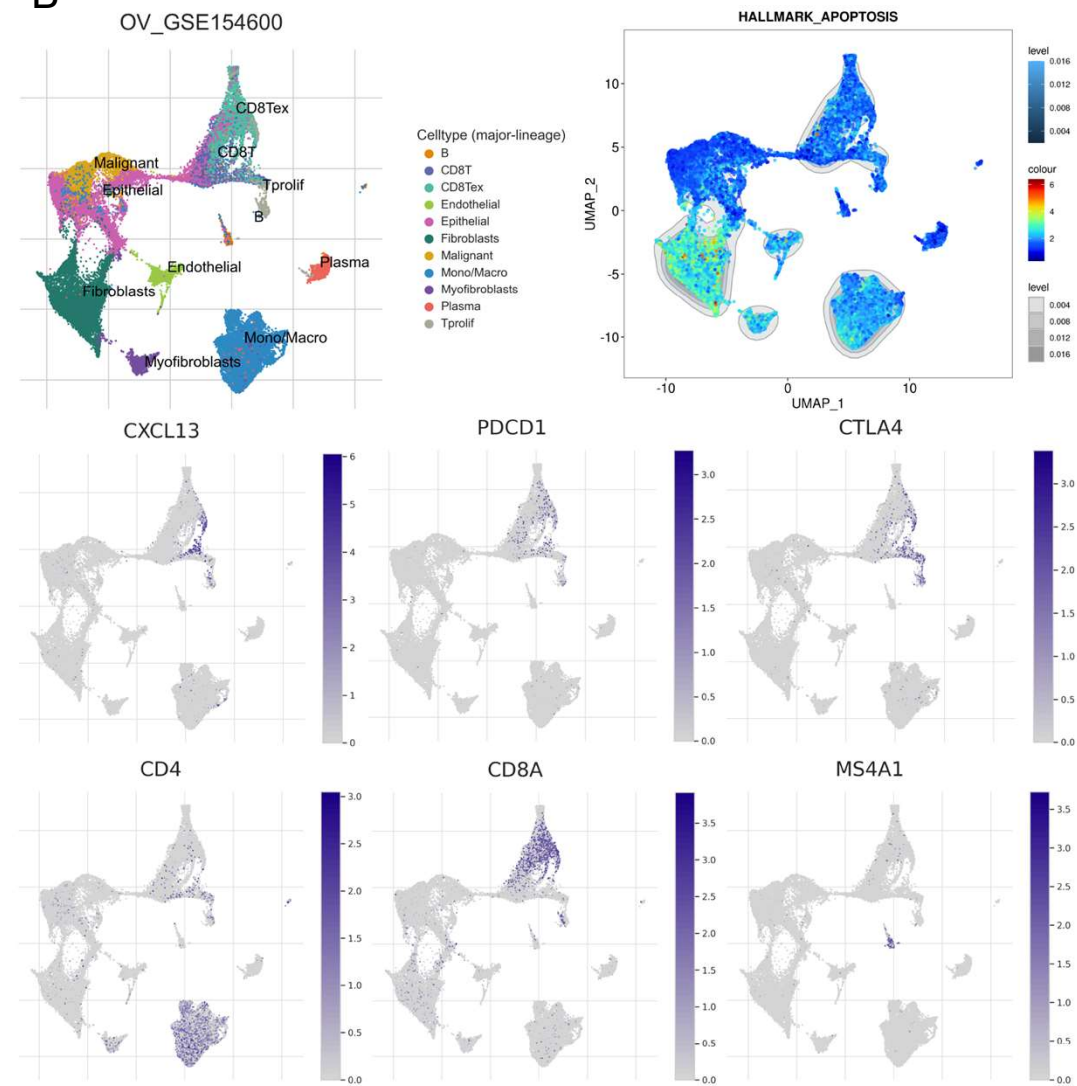

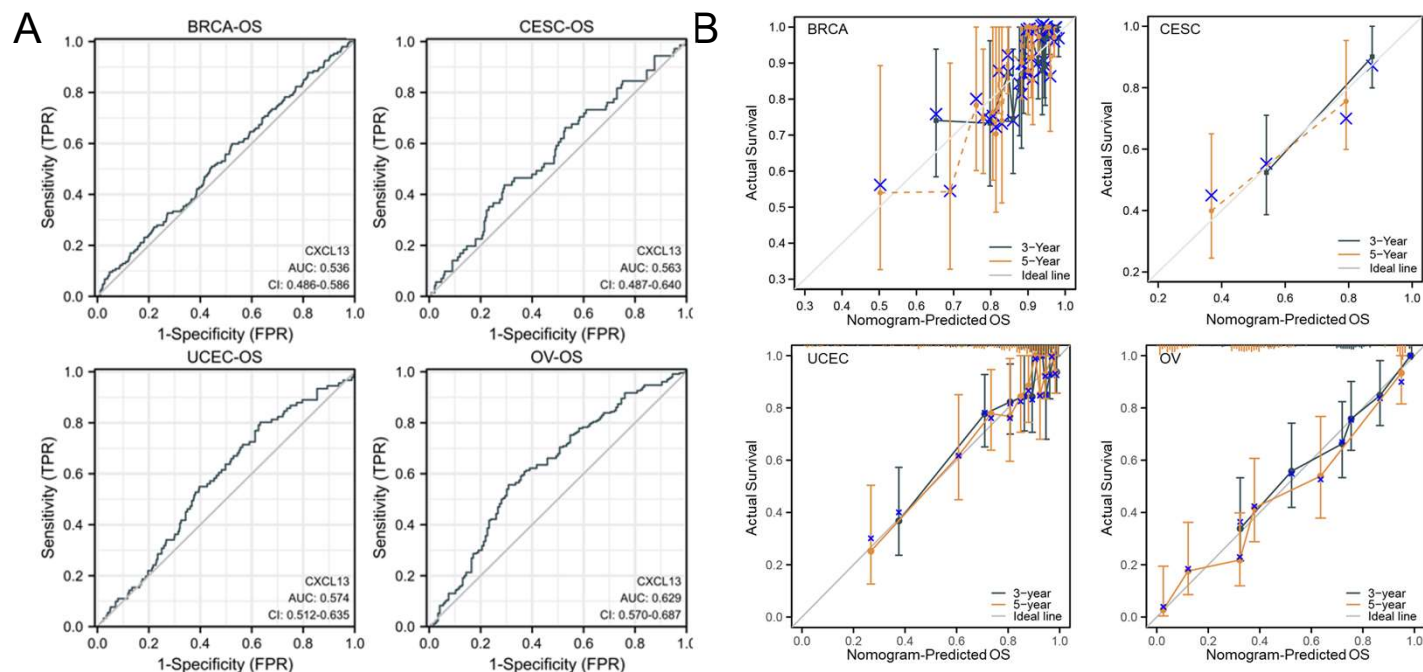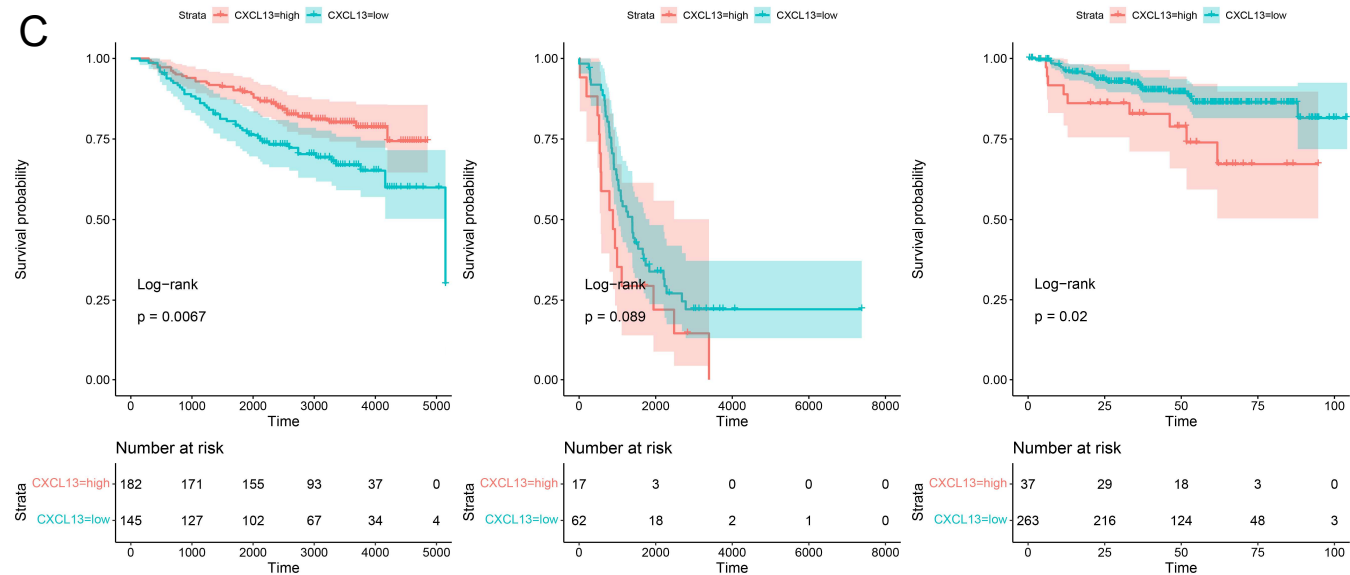

A

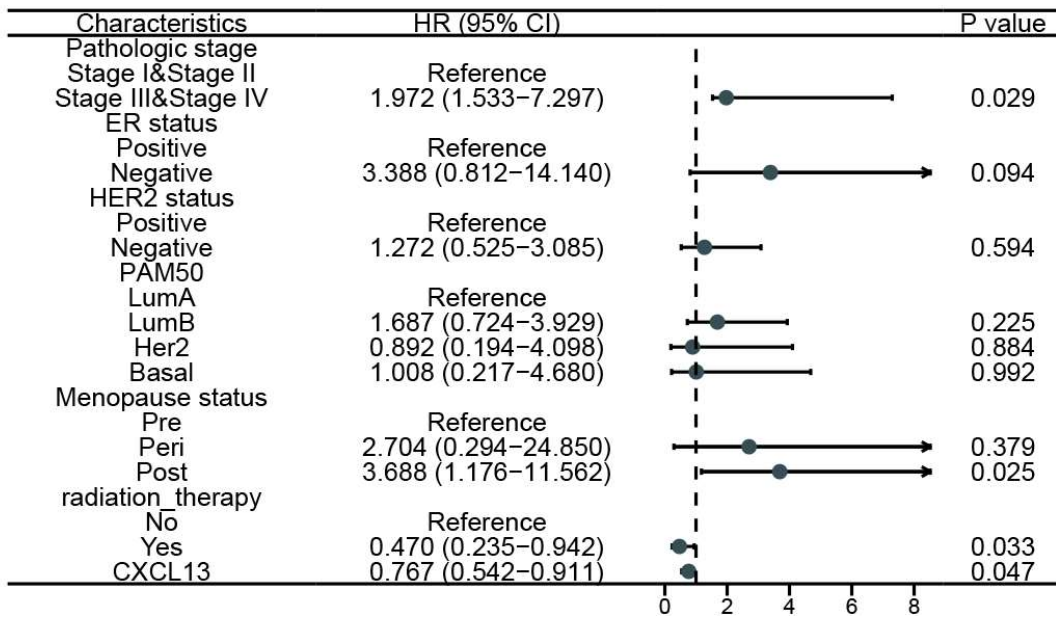

B

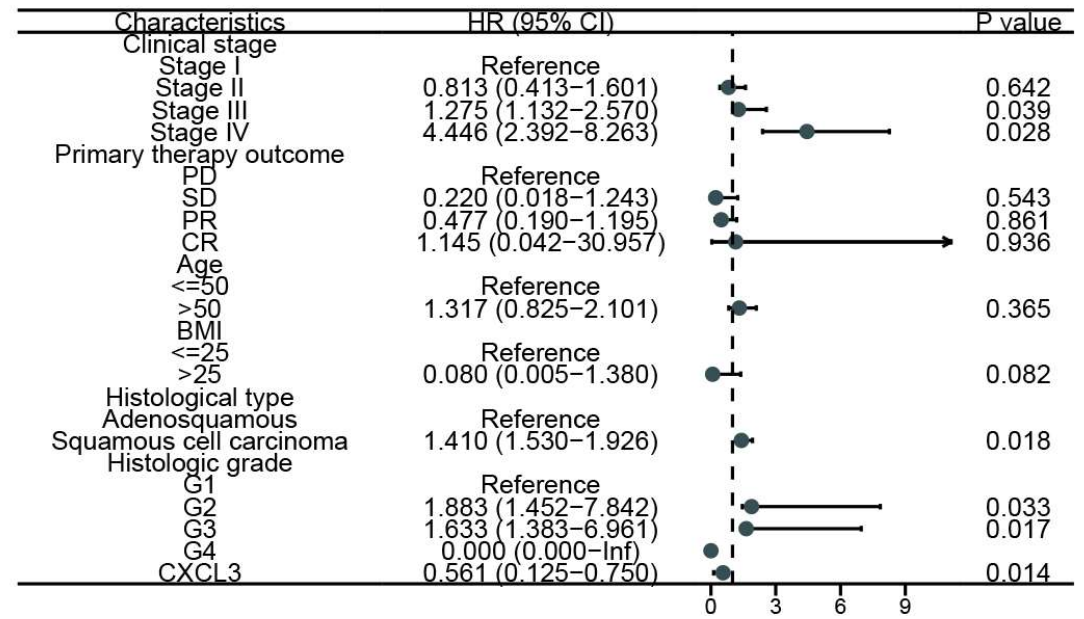

A

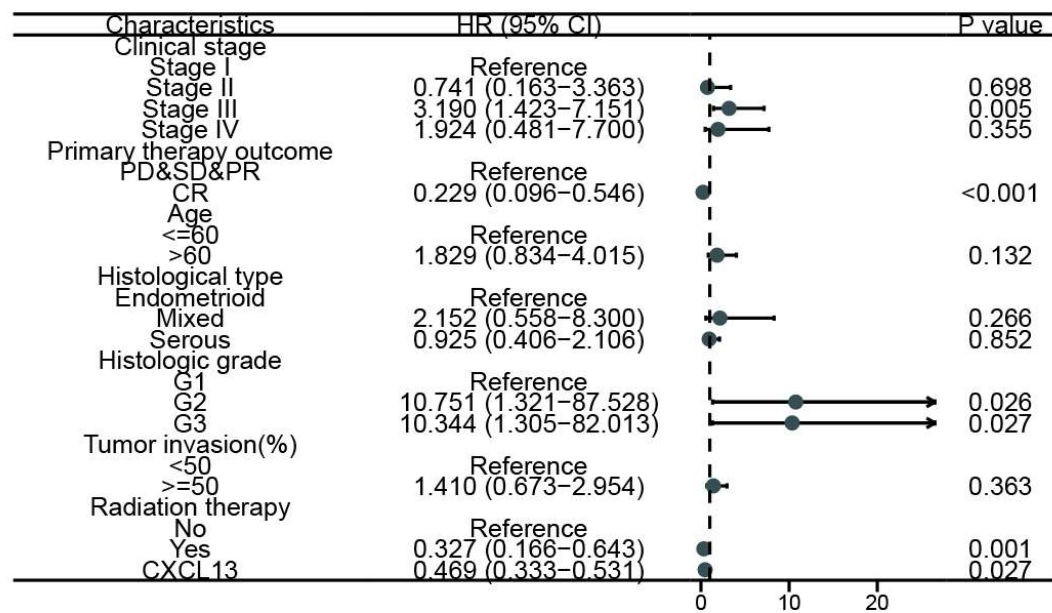

B

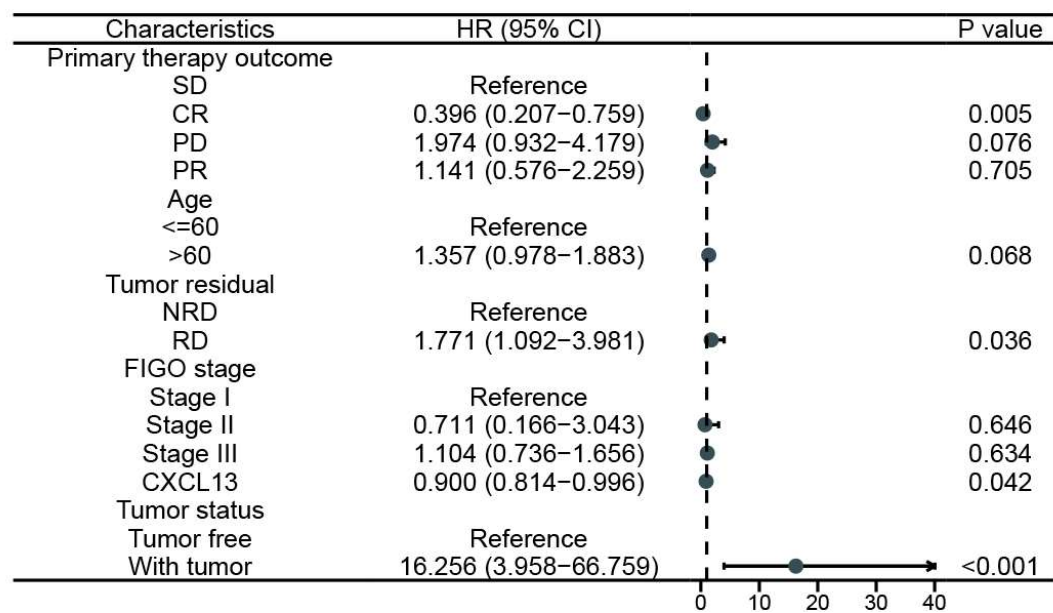

Supplement: Supplementary file 1 — Appendix A. Supplementary: Fig. S1 Top 15 most-highly mutated genes between two groups in BRCA (A) and CESC (B). Fig. S2 Top 15 most-highly mutated genes between two groups in UCEC (A) and OV (B). Fig. S3 A: The protein expression of CXCL13 in immunohistochemical images; B–C: CXCL13 expression was associated with significantly more unfavorable DSS and PFI (clockwise direction: BRCA; CESC; OV; UCEC); D: Relationship between CXCL13 and tumor stemness (left: mRNAsi; right: mDNAsi). *p < 0.05, **p < 0.01, ***p < 0.001. Fig. S4 Relationship between CXCL13 levels and immune infiltrates analyzed A: xCell; B: C IBERSORT; C: EPIC; D: QUANTISEQ; E: MCPcounter. *p < 0.05, **p < 0.01, ***p < 0.001. Fig. S5 Single-cell sequencing analyzing CXCL13 co-expression and GSEA (apoptosis pathway) analyses for BRCA (A) and CESC (B). Fig. S6 Single-cell sequencing analyzing CXCL13 co-expression and GSEA (apoptosis pathway) analyses for UCEC (A) and OV (B). Fig. S7 A: ROC curves indicating that CXCL13 could predict OS; B: Calibration curves of nomograms; C: Survival analysis of CXCL13 in GEO cohorts (left to right: BRCA; OV; UCEC). *p < 0.05, **p < 0.01, ***p < 0.001. Fig. S8 Forest plot of multivariate COX regression analysis for BRCA (A) and CESC (B). Fig. S9 Forest plot of multivariate COX regression analysis for UCEC (A) and OV (B). (PDF 4019 KB) [file 432_2024_5619_MOESM1_ESM.pdf]
